# *dizzy1* coordinates shoot and root development, constitutive stress responses, and phytohormone signaling in maize

**DOI:** 10.3389/fpls.2026.1927522

**Published:** 2026-08-05

**Authors:** Xuelian Du, Alina Klaus, Magda Alejandra Guateque Alba, Tyll Stöcker, Jorge Carvajal, Viktoria Zeisler-Diehl, Lukas Schreiber, Jeremy The, Gabriella Consonni, Heiko Schoof, Frank Hochholdinger, Caroline Marcon

**Affiliations:** 1 Crop Functional Genomics, INRES, Institute of Crop Science and Resource Conservation, University of Bonn, Bonn, Germany; 2 BonnMu: Reverse Genetic Resources, INRES, Institute of Crop Science and Resource Conservation, University of Bonn, Bonn, Germany; 3 Department of Service Platform Plant Experiments, INRES, University of Bonn, Bonn, Germany; 4 Crop Bioinformatics, INRES, Institute of Crop Science and Resource Conservation, University of Bonn, Bonn, Germany; 5 Department of Ecophysiology, IZMB, Institute of Cellular and Molecular Botany, University of Bonn, Bonn, Germany; 6 Department of Agricultural and Environmental Sciences - Production, Landscape, Agroenergy, Università degli Studi di Milano, Milan, Italy

**Keywords:** *BonnMu*, *dizzy1* mutant, lignin, maize, phytohormones, primary root, reactive oxygen species

## Abstract

We identified the monogenic recessive maize (*Zea mays* L.) mutant *dizzy1* in a segregating F_2_-family by a forward genetic screen of the *BonnMu* population, a sequence-indexed collection of *Mutator* transposon-induced mutants. *dizzy1* exhibits a dwarf phenotype with pronounced twisting of leaves and roots. Histological analyses revealed irregular cell organization in *dizzy1*, including enlarged upper epidermal cells in leaves and disorganized cortical cell architecture in roots. Physiological analysis of primary roots indicated reduced cell viability, reflected by increased membrane permeability and altered metabolic activity. Hormone response assays further showed that *dizzy1* is insensitive to brassinolide, exhibits a delayed auxin-promoted shoot response, and displays altered gibberellin effects on lateral root development. Bulked segregant RNA sequencing mapped the *dizzy1* locus to chromosome 2. Comparative transcriptome profiling of primary roots identified 4,378 differentially expressed genes between wild type and *dizzy1*, revealing widespread transcriptional reprogramming. Consistent with functional enrichment analyses, histochemical and spectrophotometric assays indicated elevated reactive oxygen species and increased lignin in *diz1* primary roots. These findings define *dizzy1* as a pleiotropic developmental mutant linking hormone signaling, redox homeostasis, and cell wall regulation in maize growth.

## Introduction

Maize dwarf mutants frequently result from defects in phytohormone biosynthesis, signaling, or transport, primarily involving gibberellin (GA), brassinosteroid (BR), and auxin. In the GA biosynthesis and signaling pathway, recessive mutants such as *anther ear1* (*an1*, Bensen et al., 1995), *dwarf1* (*d1*, Chen et al., 2014), *d3* (Winkler and Helentjaris, 1995), and *d5* (Fu et al., 2016) are impaired in GA biosynthesis, while semi-dominant mutants like *d8* and *d9* (Winkler and Freeling, 1994; Lawit et al., 2010) are defective in GA signaling. These dwarf mutants exhibit pleiotropic phenotypes including shortened internodes, broadened leaves, increased tillering, and masculinized ear florets (Robil et al., 2025). Similarly, BR biosynthesis-defective mutants, such as *nana plant1* (*na1*, Hartwig et al., 2011), *brassinosteroid-deficient dwarf1* (*brd1*, Makarevitch et al., 2012), *lilliputian1* (*lil1*, Castorina et al., 2018), and the signaling-defective mutant *gras42* (Kaur et al., 2024), consistently produce a dwarf phenotype. These mutants typically show shortened internodes, dark green, erect or twisted leaves, reduced tillering, altered leaf angle, and sterility or feminized tassels (Zhang et al., 2025). A severe dwarf phenotype, accompanied by a reduction in the number of leaves and a barren inflorescence devoid of spikelets and kernels, is also exhibited by a mutant in *vanishing tassel2* (*vt2*), a gene involved in auxin biosynthesis (Phillips et al., 2011). The auxin transport mutant *brachytic2* (*br2*; Multani et al., 2003) and the signaling mutant *brevis plant1* (*bv1*; Avila et al., 2016) also display dwarfism characterized by shortened internodes.

Independent of phytohormone-mediated pathways, dwarf phenotypes can also result from mutations in transcription factors or regulatory proteins. For instance, the *viviparous8* (*vp8*) mutant, defective in a gene encoding a putative glutamate carboxypeptidase, exhibits dwarfism with shortened internodes and narrowed leaves (Suzuki et al., 2008; Lv et al., 2014). The *crinkly4* (*cr4*) mutant, lacking a receptor-like kinase required for signaling pathways, shows dwarfism and crinkled leaves (Becraft et al., 1996). The *sucrose transporter1* (*sut1*) mutant, impaired in sucrose transport, displays dwarfism with few leaves, stunted tassel, and reduced root branching (Slewinski et al., 2009). Together, these mutants show that maize dwarfism results from perturbations in multiple developmental and metabolic pathways. It has been observed that the absence of active brassinosteroids results in altered gravitropic response of the primary root (Castorina et al., 2018). However, most studies to date have focused primarily on aerial dwarfism phenotypes, leaving the effects of these mutations on root architecture largely unexplored.

The maize root system is essential for anchorage in the soil, water and nutrient acquisition, as well as environmental adaptation (Hochholdinger, 2009). During early seedling development, the root system is primarily driven by embryonic roots, which include a single primary root and several seminal roots (Hochholdinger et al., 2018a). As the plant matures, postembryonic crown roots dominate, forming the bulk of the adult root system (Hochholdinger et al., 2018a). All root types develop postembryonic lateral roots and root hairs, which greatly expand the absorptive surface (Yu et al., 2016; Hochholdinger et al., 2018a). Anatomically, all maize roots follow a conserved structure. Longitudinally, roots feature three continuous developmental zones from the tip toward the base: the meristematic zone where cells divide, the elongation zone where cells expand, and the differentiation zone where cells specialize and form functional structures such as root hairs (Hochholdinger, 2009; Hochholdinger et al., 2018a). Radially, the root is organized into distinct concentric tissue layers, comprising the outermost epidermis, the inner cortex, and the central stele (vascular cylinder) containing xylem and phloem elements (Hochholdinger, 2009; Hochholdinger et al., 2018a). The primary root, as the first emerging organ after seed germination, plays an indispensable role in providing initial anchorage of the seedling in the soil and uptake of water and nutrients, thereby shaping the subsequent root system development. Its growth is highly sensitive to hormonal and environmental signals, making it an ideal model for dissecting the regulatory mechanisms of the root system development (Hochholdinger et al., 2018b). Therefore, understanding how dwarf mutants affect root development, especially the primary root, is essential to decipher the integrated regulatory networks of maize growth and development.

Forward genetic screens using mutagenized populations enable the discovery of potentially novel mutants, including those with altered root and/or shoot phenotypes. The *BonnMu* resource is a large maize population containing random insertions of *Mutator* (*Mu*) transposons (Marcon et al., 2020; Win et al., 2024; Marcon et al., 2025a). Sequencing of 8,064 segregating *BonnMu* F_2_-families identified 425,924 presumptive heritable *Mu* insertions, covering about 36,612 (83%) of all annotated protein coding genes in maize (B73v5; Win et al., 2024). In addition, seedling-stage images of these F_2_-families are available, which facilitates the identification of novel mutants. A major challenge in *BonnMu* forward genetics is that each F_2_-family carries many independent *Mu* insertions, making it difficult to directly link a phenotype to a single gene. On average, each family contains about 53 presumptive heritable insertions (Win et al., 2024). To reduce the number of candidate genes, bulked segregant RNA sequencing (BSR-seq) can be used (Liu et al., 2012). This method combines bulked segregant analysis (Michelmore et al., 1991) with RNA-seq to rapidly map causal genes using sequence polymorphisms such as single-nucleotide polymorphisms (SNPs). Using BSR-seq, several maize genes have been successfully cloned, including *glossy13*, which encodes a putative ABC transporter (Li et al., 2013), and *roothairless6*, which encodes a D-type cellulose synthase (Li et al., 2016).

Here, we functionally characterize a mutant designated *dizzy1* (*diz1*), isolated from the *BonnMu* resource, which exhibits dwarfism and twisted root and leaf growth. We investigate the developmental basis of primary root growth in *diz1* using an integrated approach combining morphological, histological, physiological, and transcriptomic analyses. Our results reveal multifaceted defects in the *diz1* primary root, including aberrant cell organization, reduced cell viability, increased lignin deposition and reactive oxygen species (ROS) accumulation, as well as impaired phytohormone responses. Together, these findings provide a comprehensive phenotypic characterization of *diz1* and highlight potential mechanistic frameworks underlying its root developmental defects.

## Materials and methods

### Plant materials and growth conditions

The maize *diz1* mutant was identified from the *BonnMu* F_2_-family 1-A-0328 which was generated during winter nursery of 2014–2015 in Chile, as previously described (Marcon et al., 2020; Win et al., 2024; Marcon et al., 2025a). Mutants were kept in a heterozygous state due to defects in inflorescence development. Seeds were surface-sterilized by 6% (v/v) NaClO for 5 min and then rinsed three times. They were subsequently germinated in paper rolls (Anchor Paper Co, Saint Paul, USA; Hetz et al., 1996) and incubated in a climate chamber under controlled conditions (26 °C/18 °C for 16 h light/8 h dark, 70% humidity).

### Phenotypic characterization

Seedlings were captured using a flatbed scanner (Epson expression 12000XL) and analyzed by Fiji (https://imagej.net/software/fiji/) to quantify primary root length, shoot length, and the number of seminal and crown roots. Images of primary, seminal and crown roots at seedling stage, were acquired using a stereo microscope (Leica M165 FC), and primary root hair length was subsequently quantified with Fiji. The images of seedling leaves were taken with a stereo microscope (Leica M165 FC). Images of seedling shoots, 4-week-old plants and root system were taken using a camera (SONY α6400).

### Genetic analysis

Seeds were propagated in the field nurseries at the University of Bonn (Germany), Chile, Hawaii, and Mexico in the years 2014–2025 as previously described (Marcon et al., 2020; Win et al., 2024) and subjected to successive generations of self-pollination. For backcrossing, heterozygous plants were used as male parent in crosses with different inbred lines, including B73 and Mo17. Seeds from selfed and backcrossed generations were germinated in paper rolls to assess the segregation ratios.

### Histological analyses

We examined fresh sections of leaves and roots as follows: 4–6 mm segments of fresh leaves and roots, embedded in 10% (w/v) low melt agarose (Carl Roth, Karlsruhe, Germany), were performed into 80 µm sections using a vibratome (Leica VT1200S). For root samples, the 4–6 mm region from the root hair initiation site toward the root base was excised to represent the differentiation zone. Leaf transverse sections, as well as both longitudinal and transverse sections of roots, were imaged with a dissection microscope (PALM MicroBeam, Zeiss) and analyzed by Fiji for upper epidermal cell size, leaf thickness and different tissue areas. For fresh root staining, root sections were stained with 0.2% toluidine blue O (TBO) for 30 s and rinsed three times with distilled water, followed by observation under bright-field conditions with the same microscope.

We prepared and analyzed paraffin sections as follows: differentiation zone of primary roots was collected. Samples were fixed in ice-cold formalin-aceto-alcohol (FAA) solution under vacuum to enhance tissue penetration and preservation. Following fixation, the samples were transferred into embedding cassettes, then subjected to a graded ethanol series for dehydration, and a graded Roti^®^Clear series (Carl Roth, Karlsruhe, Germany) to replace ethanol and facilitate paraffin infiltration by using an automatic tissue processor (Lecia TP1020). The cleared samples were then embedded in molten paraffin and solidified in molds at room temperature (RT). Subsequently, the paraffin blocks were trimmed and sectioned into 10 µm-sections using a rotary microtome (Lecia RM2125 RTS). The sections were mounted onto glass slides, deparaffinized in xylene, rehydrated through a descending ethanol series, and stained with 0.2% TBO for 5 min. Finally, the stained sections were dehydrated, coverslipped with a mounting medium (Carl Roth, Karlsruhe, Germany), and observed under a microscope (PALM MicroBeam, Zeiss) for structural analysis.

The fluorescent staining of primary root sections was conducted following the protocol of Ursache et al., 2018, with slight modifications. Briefly, fresh root sections were fixed in 4% (w/v) paraformaldehyde (prepared in 1 × phosphate-buffered saline (PBS)) at RT for 1 h under vacuum. Following two brief 1 min rinses in PBS buffer, the sections were moved to ClearSee solution (10% (w/v) xylitol, 15% (w/v) sodium deoxycholate, and 25% (w/v) urea) and incubated for 1 day to achieve tissue clearing. Subsequently, the sections were stained in 0.1% (w/v) calcofluor white (dissolved in ClearSee solution) for 30 min, washed in ClearSee solution for 30 min with gentle shaking, and imaged under a confocal microscopy system (Zeiss LSM 780) using 405 nm excitation with detection at 425–475 nm. For basic fuchsin staining, the clearing sections were stained overnight with 0.2% (w/v) basic fuchsin prepared in ClearSee solution. The sections were then rinsed in ClearSee solution for 1 h with gentle shaking, replacing the solution three to four times, before imaging at 543 nm excitation and 600–650 nm emission detection. Identical microscope settings were applied to image both of wild type (WT) and *diz1* mutant sections for comparability. The measurement of fluorescence intensity was performed using Fiji.

### Analysis of aerenchyma formation

The treatment under different conditions was performed according to a modified protocol (Yamauchi and Nakazono, 2022a; Yamauchi and Nakazono, 2022b). Surface-sterilized seeds were germinated in paper rolls soaked with either 2 μM DPI (Diphenyleneiodonium chloride, prepared from a 30 mM stock solution in DMSO; Sigma Chemie, Deisenhofen, Germany), 50 μM EGTA (ethylene glycol-bis(2-aminoethylether)-*N,N,N′,N′*-tetraacetic acid, from a 500 mM stock solution dissolved in 5N NaOH; Sigma Chemie, Deisenhofen, Germany), full strength nutrient solution (Hoagland’s No. 2 Basal Salt Mixture; Sigma Chemie, Deisenhofen, Germany) or distilled water (for control and O_2_ treatments). The paper rolls with seeds were placed vertically into 5 L beakers containing 1 L of the corresponding treatment solution under controlled conditions in a growth chamber. For O_2_ treatment, an aeration pump was used to continuously supply aeration into distilled water. All treatment solutions and solution-soaked paper rolls were refreshed every three days to maintain treatment effectiveness.

Root sampling was performed at two time points. At 3 days after treatment (DAT), 5 mm segments of primary root differentiation zone were collected in both WT and *diz1* mutants. At 12 DAT, 5 mm root segments were excised from the primary roots at a distance of 5 cm from the root tip in WT, and at 2 cm from the root tip in *diz1* mutants. All samples were processed for histological analysis using fresh sections as described above. The areas of aerenchyma and the root cross-sections were measured using Fiji by manually tracing their boundaries.

### Quantification of cell viability

Cell viability of primary roots was assessed using Evans blue staining and 2,3,5-triphenyltetrazolium chloride (TTC) staining. We performed the staining and quantification of Evans blue according to the method described by Vijayaraghavareddy et al., 2017 with following modifications. Briefly, primary roots were collected on ice and stained with 0.25% (w/v) Evans blue (dissolved in 0.1 M CaCl_2_ solution (pH 5.6)) for 20 min at RT in the dark. The samples were then rinsed three times with distilled water for 10 min each time before imaging. For Evans blue quantification, the stained roots were homogenized in a Tissue Lyser II (Qiagen, Hilden, Germany) with 1% (w/v) sodium dodecyl sulfate (SDS), and then incubated at 50 °C for 15 min. After centrifugation at 12,000 rpm for 15 min at RT, the absorbance of the supernatant was measured at 600 nm.

TTC staining and measurement of TTC reduction by dehydrogenase were employed as previously described (Towill and Mazur, 1975). Primary roots were harvested on ice and treated overnight with 0.4% (w/v) TTC (prepared in 0.05 M sodium phosphate buffer (pH 7.0)) at RT in the dark. Afterwards, the roots were washed three times with distilled water and then photographed. To extract the formazan product, the stained samples were ground using a Tissue Lyser II (Qiagen, Hilden, Germany), then incubated in 3 ml of 95% ethanol at 60 °C for 30 min. The extracts were then centrifuged at 12,000 rpm for 10 min at RT, and the absorbance of the supernatant was recorded at 485 nm.

### Exogenous phytohormones application

#### Brassinosteroid treatment

We conducted the exogenous epibrassinolide treatment experiment based on a modified protocol (Makarevitch et al., 2012). Briefly, surface-sterilized seeds were sown in pots containing 80 g of soil and 80 mL of ddH_2_O. The seedlings were grown under dark condition with 26 °C and 70% humidity for two days and then treated daily with 20 mL of 1 μM epibrassinolide (Sigma Chemie, Deisenhofen, Germany) solution for the next five days, followed by 30 mL of 1 μM epibrassinolide solution per day for the subsequent seven days. A 4 mM epibrassinolide stock solution was prepared by dissolving the compound in 80% (v/v) ethanol. The control group was treated in the same way with ethanol (an equivalent volume prepared in the brassinolide stock solution) diluted in ddH_2_O. 14-day-old seedlings were then collected for imaging and measurement of mesocotyl and shoot length using Fiji. The WT controls were siblings genetically matched to their respective mutants: *lil1* (derived from the selfed progeny of a *Mutator* stock outcrossed to an unrelated stock, Castorina et al., 2018) and *diz1* (from F_2_-population generated by selfing a B73 × *Mu*^4^ *per se* stock cross, Win et al., 2024; Marcon et al., 2025a).

#### Auxin and gibberellin treatment

The exogenous auxin and gibberellin application was subjected according a modified method (Hetz et al., 1996). Surface-sterilized seeds were placed onto germination paper saturated with 1 μM 1-Naphthaleneacetic acid (NAA, prepared from a 1 M stock solution in 80% (v/v) ethanol; Sigma Chemie, Deisenhofen, Germany) and 500 nM gibberellin A_3_ (GA_3_; from a 0.5 M stock solution dissolved in DMSO; Sigma Chemie, Deisenhofen, Germany). Following a 3-day incubation in darkness at 26 °C, the seedlings were moved to a growth chamber under controlled conditions for an additional 6 and 9 days. To maintain hormone activity, germinating kernels or developing seedlings were transferred to freshly prepared hormone-soaked paper and hormone solutions every 3 days. Primary root length, shoot length, and length of lateral roots formation zone were quantified using Fiji software.

### Bulked segregant RNA-seq analysis

Briefly, the F_2_ mapping population was generated by selfing F_1_ plants derived from a cross between the inbred line Mo17 and the *BonnMu* F_2_-family 1-A-0328. Primary roots (2–4 cm from the root tip) were harvested from *diz1* mutants and their WT siblings grown for 10 days. Samples were grouped into two WT pools and two *diz1* pools separately according to their phenotypes (pool 1: 60 primary roots for each phenotype; pool 2: 83 for each phenotype). Total RNA isolation from each pool was conducted using the RNeasy Plant Mini Kit (Qiagen, Hilden, Germany). RNA quality was controlled by measuring the RNA integrity number (RIN > 9) on a Bioanalyzer 2100 (Agilent Technologies, Santa Clara, CA, USA) with an Agilent RNA 6000 Nano kit (Agilent, Santa Clara, CA, USA). Finally, we constructed the RNA-seq libraries and sequenced on the NovaSeq 6000 platform (Novogene, Cambridge, UK) with a paired-end 150 bp (PE150). After sequencing, the R package QTLseqr was used to analyze the SNP data following the method described by Mansfeld and Grumet, 2018. A G’ analysis with a smoothing window size of 3.5 Mb was performed (Magwene et al., 2011), and the resulting SNP distribution and G’ values were plotted against their corresponding genomic position (Mb).

### RNA isolation and RNA-sequencing

Primary roots of WT at 6 days after germination (DAG) and *diz1* seedlings at 9 DAG, which had comparable primary root lengths (~4 cm), were pooled in three biological replicates, respectively. Each replicate contained 4–5 primary roots. Subsequently, the samples were immediately frozen in liquid nitrogen for RNA isolation. RNA isolation was performed using the RNeasy Plant Mini Kit (Qiagen, Hilden, Germany) according to the instructions of manufacturer. The RNA samples were treated with RNase-Free DNase (Qiagen, Hilden, Germany) to avoid DNA contamination. Quality and integrity of total RNA were evaluated using a NanoDrop (Thermo Fisher Scientific, Waltham, MA, USA) and a Bioanalyzer (Agilent Technologies, Santa Clara, CA, USA) with the Agilent RNA 6000 Nano kit (Agilent, Santa Clara, CA, USA). For all RNA samples, RNA integrity number values were ≥ 7. A paired-end mRNA sequencing of samples was performed on a NovaSeq 6000 PE150 platform (Novogene, Cambridge, UK).

### Processing of RNA-seq raw data

Raw reads were trimmed with trimmomatic (version 0.39; Bolger et al., 2014) in paired-end mode with the parameters of ILLUMINACLIP:adapter.fa:2:30:10 LEADING:3 TRAILING:3 SLIDINGWINDOW:4:15 MINLEN:60. Trimmed reads < 60 bp were discarded, and the remaining reads were aligned to the maize B73v5 reference genome (https://download.maizegdb.org/Zm-B73-REFERENCE-NAM-5.0/) with HISAT2 (version 2.2.1; Kim et al., 2015). Read quantification was conducted with featureCounts (Liao et al., 2014). Only reads uniquely aligned to the reference genome were considered for further analysis. Principal component analysis was conducted in R with the prcomp() function, and the resulting components were visualized with the autoplot() function from the ggplot2 package.

### Statistical assessment of differentially expressed genes

Inactive and lowly expressed genes were excluded by applying a counts per million (CPM) cutoff of 0.4, and the remaining read counts were normalized for sequencing depth with the cpm() function implemented in the edgeR package (Robinson et al., 2010). Prior to differential expression analysis, the data underwent log2 transformation. A linear model with a fixed effect for the factor genotype was fitted to the dataset. To account for mean-variance dependencies, the voom() function from the limma package (Ritchie et al., 2015) was employed, enabling the estimation of precision weights to adjust for heteroscedasticity. The voom transformed count matrix was subsequently processed with the eBayes() function to estimate overall variability of genes and shrink the standard errors toward a global value. Differentially expressed genes (DEGs) were identified through pairwise comparison between WT and *diz1* samples using the decideTests() function. The *p*-values were adjusted for multiplicity with a false discovery rate (FDR) < 5% (Benjamini and Hochberg, 1995). All gene models that exhibited a |log_2_ fold change| (|log_2_FC|) > 1 and FDR < 5% were classified as differentially expressed.

### Analysis of gene ontology term enrichment

GO terms were assigned to DEGs using the topGO R package to carry out an enrichment analysis (Alexa et al., 2006). To refine the analysis of regulatory mechanisms, the DEGs were categorized based on their direction of regulation before conducting the enrichment analysis. Functional enrichment was calculated with a Fisher’s exact test based on the weight01 algorithm to reduce the number of false positives.

### Detection and quantification of hydrogen peroxide (H_2_O_2_) and superoxide radical (O_2_^-^)

Reactive oxygen species (ROS) levels in primary roots of WT and *diz1* mutant were evaluated through histochemical staining with 3,3′-diaminobenzidine (DAB) and nitroblue tetrazolium (NBT), followed by spectrophotometric analysis. To visualize H_2_O_2_, excised primary roots were placed in 0.1% (w/v) DAB staining, initially dissolved in dH_2_O at pH 3.8 and subsequently adjusted to pH 7.5 with 0.05% (v/v) Tween 20 and 5% (v/v) 200 mM Na_2_HPO_4_, based on a modified protocol (Daudi and O’brien, 2012). Roots were vacuum-infiltrated to ensure complete dye uptake and then incubated without vacuum in the dark for 8–12 h, then washed thoroughly with distilled water to remove excess reagent before imaging. For H_2_O_2_ quantification, a protocol modified from Kotchoni et al., 2006 was used. DAB-treated roots were ground in 0.2 M HClO_4_ using a Tissue Lyser II (Qiagen, Hilden, Germany) and then centrifuged at 12,000 rpm for 10 min at 4 °C. The absorbance of the resulting supernatant was immediately measured at 450 nm. The H_2_O_2_ concentrations were calculated using a standard curve generated with known amounts of H_2_O_2_ in 0.2 M HClO_4_-DAB.

A modified method for O_2_^-^ detection and quantification was followed by Grellet Bournonville and Díaz‐Ricci, 2011. Whole primary roots were immersed with 0.05% (w/v) NBT prepared in 0.05 M sodium phosphate buffer (pH 7.0). The roots were first subjected to vacuum infiltration to enhance reagent penetration and then incubated without vacuum for 2–4 h in the dark. Following staining, roots were rinsed with distilled water for 3 times to remove unbound NBT and subsequently photographed. For quantification, NBT-stained roots were homogenized in Tissue Lyser II (Qiagen, Hilden, Germany). The resulting powder was extracted in 2 M KOH-DMSO (1/1.16; v/v) and then centrifuged for 10 min at 12,000 rpm. Absorbance of the supernatant was recorded at 630 nm and compared to a standard curve constructed with known concentrations of NBT in the 2 M KOH-DMSO mixture.

### Quantification of lignin content

For comparative analysis, we prepared three sample groups: *diz1* at 9 DAG, WT at 5 DAG and WT at 9 DAG. For each group, primary roots with lateral roots removed from twenty seedlings were equally pooled into four biological replicates. Prior to analysis, samples were enzymatically digested with 0.5% (w/v) cellulase and 0.5% (w/v) pectinase at room temperature under gentle shaking (Zeier and Schreiber, 1997). The enzyme solution was changed every 2–3 days for two weeks. Digested roots were washed with borax buffer, then deionized water and at last transferred to chloroform:methanol (1:1, v/v) to remove all soluble lipids. Subsequently, the root samples were dried, weighed and cut into very fine pieces (Baales et al., 2021). 1–2 mg of the dried samples were incubated in thioacidolysis reagent for 4 hours in autosampler vials (Foster et al., 2010). The samples were vortexed once every hour, making sure that the sample material remained constantly in the reagent throughout the digestion.

Upon completion of the reaction, vials containing the samples were cooled to room temperature, and spiked with 10 µg of C32 (dotriacontane) as an internal standard. To stop the reaction, 500 µL of saturated NaHCO_3_ solution was added. Afterwards, samples were extracted three times with 1 mL ethyl acetate. The combined organic extracts were dried in a heating block and 500 µL of acetone were applied twice to remove excess water at 60 °C under a mild nitrogen stream.

For lignin monomer analysis by gas chromatography (GC), samples were derivatized with 20 µL pyridine and 100 µL N,O‐bis(trimethylsilyl)acetamide (BSA; Sigma Chemie, Deisenhofen, Germany) reagent for 45 min at 70 °C. Lignin monomers were quantified by injecting 1 µL of sample on a splitter system on a gas chromatography connected to flame ionization detection (GC–FID; HP6890 N, Hewlett-Packard). Monomers were identified by gas chromatography connected to mass spectrometry (GC–MS; 5977B, Agilent). The obtained mass spectra of lignin monomers were identified according to Rolando et al., 1992 thioacidolysis products.

## Results

### Isolation and genetic characterization of the *diz1* mutant

We isolated the *diz1* mutant in a forward genetic screen of 8,064 segregating F_2_-families from our in-house *BonnMu* transposon-induced maize resource to identify mutants with altered seedling shoot or root development. We germinated seeds using a paper roll system and screened seedlings for visible non-wild type (WT) phenotypes 9–12 days after germination (DAG; Marcon et al., 2025b). From this screen, we identified a dwarf mutant exhibiting twisted roots and leaves in the F_2_-family *BonnMu*-1-A-0328 and designated it *dizzy1* (*diz1*). A segregation analysis of this *BonnMu* F_2_-family revealed a 3.6:1 (WT:mutant) ratio, approximating the 3:1 ratio expected for a monogenic recessive mutant (**Supplementary Table 1**). The *diz1* mutants developed feminized tassels, thus could not be selfed. Therefore, we maintained the mutant by selfing heterozygous WT plants for several generations, consistently generating WT and mutant seedlings in an approximately 3:1 ratio (**Supplementary Table 1**). Heterozygous WT plants were also introgressed into the inbred lines B73 and Mo17. All backcrosses produced *diz1* phenotypes in the F_2_-progeny, with segregation patterns statistically consistent with Mendelian expectations, as confirmed by χ^2^ tests (**Supplementary Table 1**). Together, these results demonstrate that *diz1* segregates as a monogenic recessive mutation.

### The *diz1* mutant exhibits pleiotropic phenotypic effects

**Figure 1:**
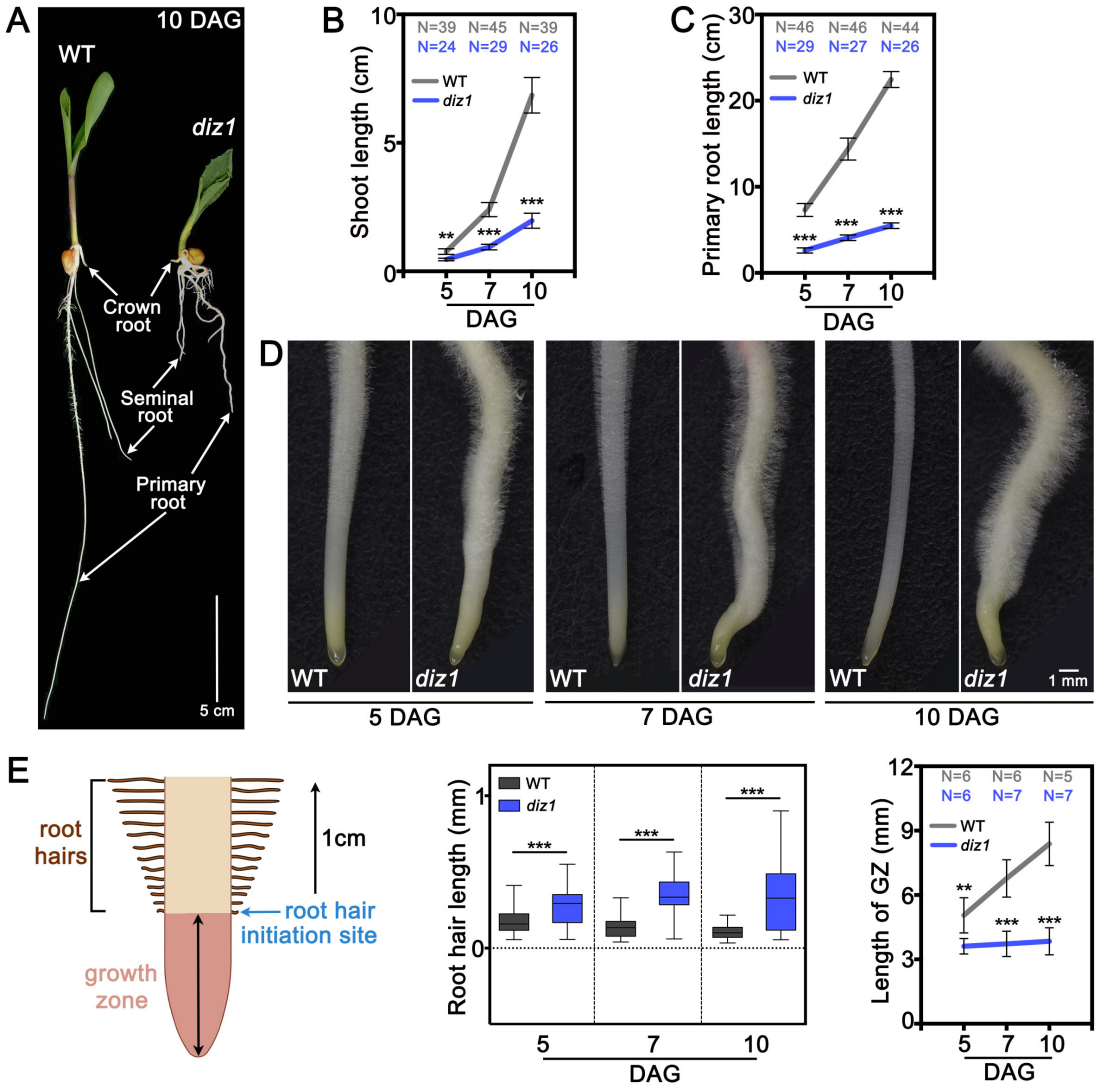
Phenotypic properties of the *dizzy1* (*diz1*) mutant. **(A)** Wild type (WT) and *diz1* seedlings at 12 days after germination (DAG). Arrows indicate different root types. Scale bar = 5 cm. **(B)** Shoot length at 5, 7, and 10 DAG. **(C)** Primary root length at 5, 7, and 10 DAG. Sample sizes in **(B, C)** are shown above the columns. Error bars in **(B)** and **(C)** represent 95% confidence interval. **(D)** Primary roots imaged under a stereo microscope at 5, 7, and 10 DAG. Scale bar for all images in this panel = 1 mm. **(E)** Left: schematic of analyzed primary root regions, showing the root hair zone (first 1 cm from initiation site) and growth zone (distance from root tip to root hair initiation site, including meristematic and elongation zone). Middle: root hair length at 5, 7, and 10 DAG (n = 6–7 roots/genotype). The center line of the boxplots indicates the median, the box limits indicate the 25th and 75th percentiles, and the whiskers extend to the minimum and maximum values. Right: growth zone (GZ) length at the same stages, sample sizes above columns. Error bars represent 95% confidence interval. Asterisks in **(B, C, E)** indicate significant differences based on Welch’s *t*-test: **p < 0.01; ***p < 0.001.

To characterize the morphology of the *diz1* mutant in detail, we germinated segregating seeds and examined the seedlings phenotypically. Compared to WT siblings, *diz1* mutants exhibited shortened and twisted leaves and roots (**Figures 1A, D**; **Supplementary Figure 1A**). Already at 5 DAG, the *diz1* shoots, as measured from the kernel to the tip of the coleoptile, were significantly reduced by 40% compared to the WT (**Figure 1B**). Similarly, at 5 DAG, *diz1* primary roots were significantly shorter by 64% (**Figure 1C**). Notably, *diz1* seedlings continued to exhibit markedly reduced shoot and root lengths at both 7 and 10 DAG, with shoot lengths reduced by 61% and 71%, and primary root lengths reduced by 71% and 76%, respectively (**Figures 1B, C**). These results suggest that the *diz1* mutation strongly and persistently inhibits both shoot and primary root growth during seedling development. Comparative analysis of 4-week-old WT and *diz1* mutants grown in pots further revealed the dwarfed stature and the distorted leaf shape in the mutant (**Supplementary Figure 1B**). Similarly, the overall root system of the mutants was reduced (**Supplementary Figure 1C**). In the field, *diz1* mutants exhibited a feminized tassel and shortened internodes at the reproductive stage (**Supplementary Figure 1D**). These observations further support the pleiotropic effects of the *diz1* mutation across the vegetative, reproductive and maturity stages.

Compared to WT, the mutant primary root showed progressively twisted growth, longer root hairs, and a shortened growth zone comprising the meristematic and elongation zones (**Figure 1D**). We subsequently quantified these observations by measuring the length of root hairs and growth zone. Specifically, we measured root hair length within a 1 cm region from the root hair initiation site toward the kernel. We defined the growth zone as the distance from the root tip to the root hair initiation site, encompassing both the meristematic and elongation zones (**Figure 1E**). This analysis revealed that root hair length increased markedly in the mutants, with an average increase of about 66% at 5 DAG, 121% at 7 DAG, and 285% at 10 DAG (**Figure 1E**). In contrast, the length of the growth zone in the mutants was reduced by about 26% at 5 DAG, 45% at 7 DAG, and 54% at 10 DAG (**Figure 1E**). Together, these results indicate that the *diz1* mutation causes pleiotropic defects in both shoot and root architecture, with strong effects on root growth zone and root hair elongation during seedling development.

### Anatomical defects in leaf blade and primary root tissues of the *diz1* mutant

**Figure 2:**
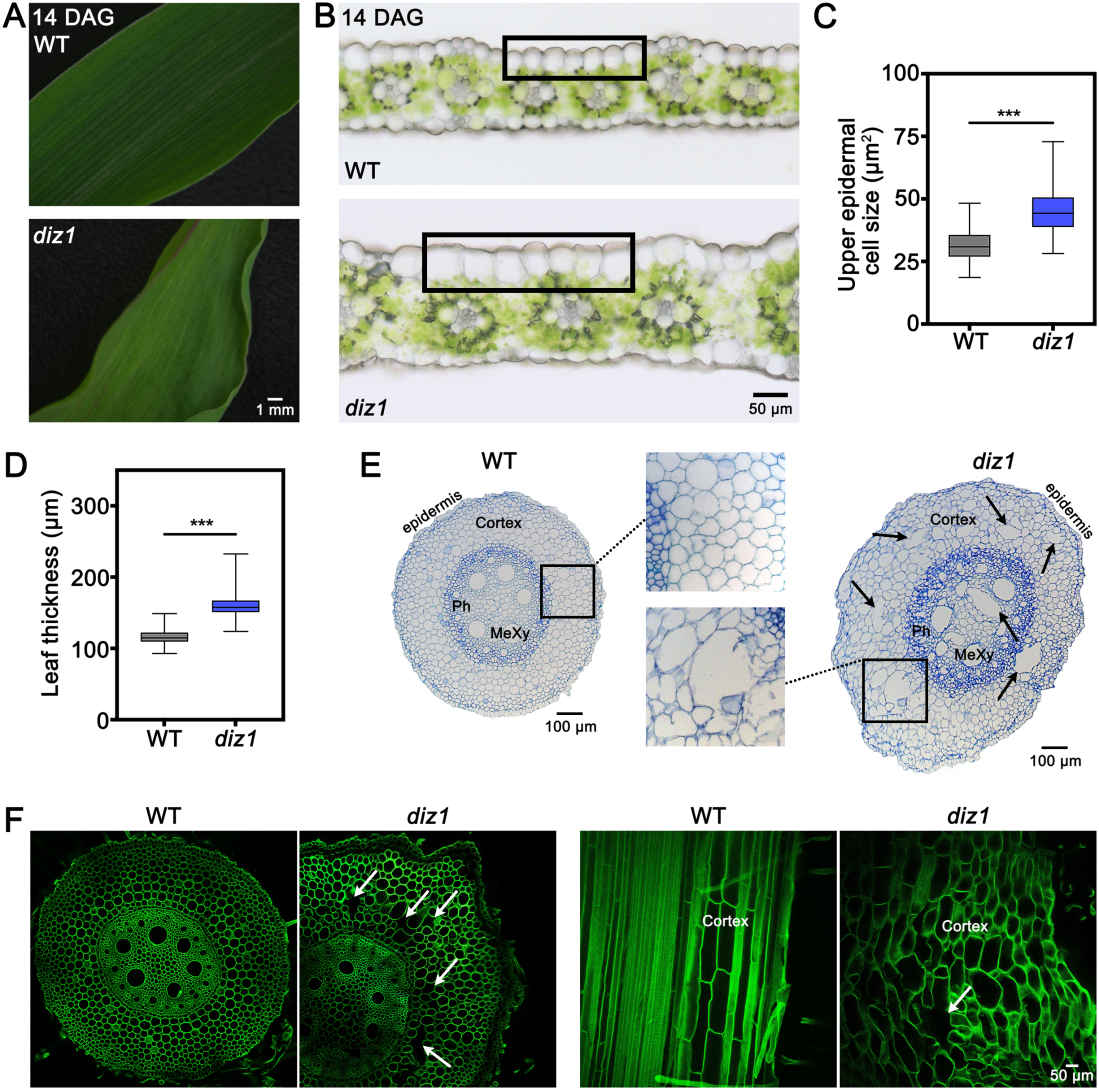
Histological analyses of the *diz1* mutant. **(A)** Close-up of WT and *diz1* leaves at 14 DAG. Scale bar for both images = 1 mm. **(B)** Fresh leaf cross-sections at 14 DAG. Black boxes highlight seven epidermal cells of WT and *diz1*, respectively. Scale bar for both images = 50 μm. **(C, D)** Quantification of upper epidermal cell size **(C)** and leaf thickness **(D)** in WT and *diz1* at 14 DAG. Data were collected from 3–4 leaves of 4–5 seedlings per genotype. The center line of the boxplots indicates the median, the box limits indicate the 25th and 75th percentiles, and the whiskers extend to the minimum and maximum values. Significant differences were calculated by Welch’s *t*-test: ***p < 0.001. **(E)** Paraffin cross-sections of the primary root differentiation zone at 3 DAG, stained with toluidine blue O (TBO). Zoom-in (dashed line) shows area within black box. Black arrows show the enlarged cells or expanded intercellular spaces in *diz1*. Ph: phloem. MeXy: metaxylem. Scale bars are included in each image. **(F)** Confocal images of cross- (left) and longitudinal sections (right) of differentiation zone in the primary root of WT and *diz1* at 3 DAG, stained with calcofluor white. White arrows indicate intercellular spaces within the cortical parenchyma of *diz1*. Scale bar for all images in this panel = 50 μm.

Similar to the young root system, the leaf blade of *diz1* displayed helical upward and inward curling, along with an undulated surface texture (**Figure 2A**; **Supplementary Figure 1A**). To determine whether these phenotypic defects are associated with alterations in tissue organization and cellular architecture, we performed a comparative histological analysis of WT and *diz1* seedling leaves and roots. Cross-sections of mutant leaf blades showed that overall tissue organization, including epidermis, mesophyll, sclerenchyma, bundle sheath, and vasculature, was similar to the WT (**Figure 2B**). However, the upper epidermal cells of *diz1* were enlarged (**Figure 2B**). Quantification confirmed an average upper epidermal cell size of 45 µm² in the mutant versus 31 µm² in the WT (**Figure 2C**). Leaf thickness was also increased by 38% in the mutant (**Figure 2D**). These alterations in epidermal cell size and leaf thickness could contribute to the helical leaf morphology of the mutant.

We stained transverse paraffin sections of the differentiation zone in primary roots from both genotypes at 3 DAG with toluidine blue O (TBO). TBO is a polychromatic dye that differentially stains cell wall components, such as lignin in blue and carboxylated polysaccharides in purple (Bellinger et al., 2019). We chose 3 DAG for this analysis, as the mutant develops, i.e., the *diz1* primary root becomes increasingly twisted, making transverse sectioning challenging at later developmental stages. Moreover, 3 DAG represents the earliest time point at which the mutant phenotype is clearly manifested. The WT primary roots displayed a typical radial pattern, characterized by multiple layers of outer cortical parenchyma surrounding the central cylinder with well-organized xylem and phloem elements (**Figure 2E**). In contrast, while the *diz1* mutant retained an overall radial pattern similar to the WT, the metaxylem within the vascular tissue and a subset of cortical cells showed disorganization, including either enlarged cells or expanded intercellular spaces (**Figure 2E**). To assess whether enlarged cells or rather expanded intercellular spaces cause the disarrangement, we prepared fresh transverse and longitudinal sections of primary roots in WT and *diz1* at 3 DAG, followed by calcofluor white staining. Calcofluor white is a fluorescent dye that binds specifically to cell walls and thus highlights cellular boundaries (Ursache et al., 2018). In cross-sections, the mutant displayed a loss of compact, concentric cortical organization characteristic of the WT, along with irregular cortical intercellular spaces (**Figure 2F**). Notably, we did not observe the irregular intercellular spaces or disorganized metaxylem in the vascular tissue of mutants shown in **Figure 2E** in these preparations. This feature may appear only in severe phenotypes. In addition, the epidermis of the mutant formed a jagged outline, deviating from the smooth and regular arrangement in the WT (**Figure 2F**). Longitudinal sections further revealed that, unlike the uniformly elongated cortical cells of the WT, *diz1* exhibited undulated and pleiomorphic cortical cells (**Figure 2F**).

To further examine the spatial pattern of root defects, we prepared fresh longitudinal and serial transverse sections of primary roots at 3 DAG and stained them with TBO. Both section types revealed tissue defects in *diz1* already in the early elongation zone (**Supplementary Figure 2A**). In the differentiation zone, cells in longitudinal sections lost their elongated shape, resulting in a wavy root structure, and cortical spaces appeared discontinuous along the root (**Supplementary Figure 2A**). We subsequently quantified the cross-sectional areas in the meristematic, elongation, and differentiation zones. Compared to WT, the differentiation and elongation zones in the mutant showed 40–44% increases in cross-sectional cortex and stele areas, while the meristematic zone exhibited smaller but consistent increases of 21–33% (**Supplementary Figure 2B**). These structural defects support the hypothesis that the reduced elongation capacity and twisted root growth of the mutant result from impaired cell proliferation, expansion and differentiation during early seedling development.

### Consistent tissue defects and developmental delay in seminal and crown roots of *diz1*

We also assessed seminal and crown root development in WT and *diz1* mutants. At 5, 7, and 10 DAG, seminal roots were present in approximately 50%, 100%, and 100% of WT plants, respectively, compared with 0%, 39%, and 77% of mutants (**Supplementary Figure 3A**). At 10 DAG, most WT possessed three seminal roots, while 23% of the mutants lacked seminal roots and 39% had only two (**Supplementary Figure 3A**). Similarly, 33% and 82% of the WT developed crown roots at 10 and 12 DAG, respectively, with 9% individuals producing up to four roots (**Supplementary Figure 3A**). In contrast, only 8% and 73% of the mutants formed crown roots at 10 and 12 DAG, respectively (**Supplementary Figure 3A**). At these developmental stages, the majority of *diz1* mutants produced only a single crown root (**Supplementary Figure 3A**). These findings indicate a delayed development and reduced numbers of both seminal and crown roots in *diz1* mutants. Moreover, helically twisted growth, elongated root hairs, and a shortened growth zone were also observed in the seminal and crown roots of the mutants (**Supplementary Figures 3B, C**), consistent with the phenotypes of the primary root. Fresh cross-sections of the differentiation zone in seminal and crown roots at 12 DAG also revealed disorganized cortical cells and irregular intercellular spaces in the *diz1* cortex (**Supplementary Figures 3D, E**). Taken together, these results show that cellular disorganization and helical twisting are consistent features throughout the root system of *diz1* mutants.

### Cortical intercellular spaces in the *diz1* mutant are not lysigenous aerenchyma

The intercellular spaces observed in *diz1* roots could indicate the presence of lysigenous aerenchyma. In the maize root, lysigenous aerenchyma is an air space formed through programmed cell death of cortical cells under oxygen- or nutrient-deficiency, which enables efficient oxygen diffusion (Yamauchi and Nakazono, 2022a). This process is known to be regulated by ethylene, ROS, and calcium (Ca²^+^) signaling molecules (Yamauchi et al., 2013; Yamauchi and Nakazono, 2022a). The ethylene-ROS-Ca^2+^ pathway can be chemically inhibited or stimulated (Yamauchi et al., 2013; Yamauchi and Nakazono, 2022a). To determine whether the intercellular spaces in the cortical parenchyma of *diz1* represent true aerenchyma, we subjected both WT and mutant plants to a series of inhibitor and environmental interventions targeting the pathway of aerenchyma formation. Specifically, we used diphenyleneiodonium (DPI), an inhibitor of the respiratory burst oxidase homolog (RBOH), to block RBOH-derived ROS production. We also applied ethylene glycol-bis(2-aminoethylether)-*N,N,N′,N′*-tetraacetic acid (EGTA), a Ca²^+^ chelator that limits Ca^2+^ influx into the cytosol. Moreover, we grew plants under aerated conditions or a full-strength nutrient solution to rule out aerenchyma induction by oxygen or nutrient deprivation. After 3 days of treatment (DAT), we examined transverse sections for the differentiation zone of primary roots. No aerenchyma formation was observed under these conditions in WT, whereas the mutant developed irregular cortical spaces regardless of the treatment, indicating that these aerenchyma-inhibiting conditions did not affect cortical spaces formation in *diz1* mutant (**Supplementary Figure 4A**). To verify whether these treatments effectively suppress aerenchyma formation, we further examined transverse sections at 12 DAT. In WT roots, all treatments reduced aerenchyma formation compared to the control (**Supplementary Figures 4B, C**), confirming their inhibitory effect. In contrast, *diz1* did not form aerenchyma at this stage, and instead consistently displayed irregular cortical spaces across all treatments (**Supplementary Figure 4B**). Notably, under these conditions that clearly suppress aerenchyma in WT, the area of intercellular spaces in *diz1* mutants showed no significant differences compared to the control (**Supplementary Figure 4D**). Moreover, in contrast to root aerenchyma, which typically develops in a longitudinally continuous pattern and strictly cortical localization, the intercellular spaces in the mutant were irregular, longitudinal discontinuous, and randomly distributed, occurring mostly within the cortex but occasionally within the stele, as exemplified in the *diz1* mutant with DPI treatment (**Supplementary Figure 4B**). These findings demonstrate that the cortical spaces in the mutant root are different from lysigenous aerenchyma.

### Decreased cell viability in the primary root of *diz1*

To assess whether cell viability is altered in primary roots of *diz1* seedlings, we performed Evans blue and 2,3,5-triphenyltetrazolium chloride (TTC) staining on WT and mutant plants (**Supplementary Figure 5A**). Evans blue selectively penetrates and imparts blue staining to cells with impaired membranes (Vijayaraghavareddy et al., 2017). TTC is reduced by mitochondrial dehydrogenases to form insoluble red formazan, a reaction that distinguishes metabolically active cells (Lopez Del Egido et al., 2017). To account for the growth inhibition and altered root morphology (development) in the *diz1* mutant, we stained primary roots both at comparable developmental stages (5, 7, and 9 DAG) and at an equivalent root length (9 cm).

Evans blue staining of WT roots gradually decreased in the elongation zone compared to the root tip but increased in the differentiation zone over time (**Supplementary Figure 5A**). In contrast, *diz1* roots showed progressively stronger staining across the entire root with age, except for a transient reduction in part of the elongation zone at 7 DAG (**Supplementary Figure 5A**). At all stages, including in roots of equal length (9 cm), mutant roots stained more intensely than WT (**Supplementary Figure 5A**). Spectrophotometric quantification of Evans blue uptake, normalized to root surface area, confirmed higher dye accumulation in *diz1* than WT at every time point (**Supplementary Figure 5C**). WT roots showed a developmental decline in dye uptake (**Supplementary Figure 5C**), indicating progressive improvement in membrane integrity. In contrast, *diz1* roots exhibited a transient reduction in Evans blue uptake at 7 DAG, followed by an increase at 9 DAG, suggesting a loss of membrane stability. Moreover, even when roots of identical length were compared, *diz1* showed higher Evans blue uptake (**Supplementary Figure 5C**). Together, these results indicate that primary root cell membranes of *diz1* mutants lose integrity already at the early seedling stage.

TTC staining produced consistent results. Staining intensity declined with age in WT roots but increased in *diz1* (**Supplementary Figure 5B**). Quantification of reduced formazan accumulation, normalized to surface area, decreased over time in both genotypes, but more steeply in *diz1* (**Supplementary Figure 5D**), likely due to impaired root growth dynamics. Mutant roots consistently showed stronger TTC staining and higher formazan accumulation than WT (**Supplementary Figures 5B,D**). Interestingly, 9 cm-long *diz1* roots stained more strongly than WT roots of the same length (**Supplementary Figure 5B**), yet accumulating less formazan per surface area (**Supplementary Figure 5D**). This apparent discrepancy reflects developmental timing: 9 cm WT roots were sampled at 4–5 DAG, when formazan content peaked, whereas 9 cm *diz1* roots were sampled at 10–12 DAG, when formazan levels had already declined. The difference could have been further amplified by surface area dilution in the *diz1* mutant. Together, these findings demonstrate that the *diz1* roots exhibit early and persistent defects in cell viability, reflected in increased membrane permeability and metabolic activity.

### A brassinosteroid-insensitive pathway underlies the dwarf phenotype of *diz1*

The stunted shoots, crinkled leaves, and sterility of adult *diz1* plants resemble phenotypes seen in maize brassinosteroid biosynthesis mutants such as *brd1* (Makarevitch et al., 2012) and its allele *lil1* (Castorina et al., 2018). Given the phenotypic similarity, we tested whether *diz1* is allelic to *lil1* by crossing heterozygous *diz1* with heterozygous *lil1* plants. While all plants in the F_1_-population displayed the WT phenotype, the F_2_-population exhibited a phenotypic segregation ratio of 9:3:3:1 (not shown), indicating that *diz1* is not allelic to *lil1*. Consistent with this, *diz1* seedlings display twisted leaves and a crinkled root-dwarf phenotype, whereas *lil1* seedlings have smooth leaves and straight-growing roots. At maturity, *diz1* leaves are pale green, unlike the dark green leaves of *lil1* (Castorina et al., 2018).

We next tested whether the *diz1* shoot phenotype could be rescued by exogenous brassinolide (BL), as previously shown for *brd1* (Makarevitch et al., 2012). To this end, *lil1* and *diz1* mutants and their WT siblings were grown in soil in darkness for two weeks with or without 1 μM BL. WT siblings of *lil1* and *diz1* exhibited a typical etiolation response (**Supplementary Figures 6A, D**), with mesocotyl elongation reaching about 8 cm (*lil1*) and 6 cm (*diz1*; **Supplementary Figures 6B, E**). Exogenous BL application significantly reduced mesocotyl elongation in the WT by 10% (*lil1* siblings) and 19% (*diz1* siblings; **Supplementary Figures 6B, E**), and also reduced shoot length by 13% and 10% (**Supplementary Figures 6C, F**), respectively. Shoot length was measured from the first shoot node (the node from which the first whorl of crown roots emerges) to the tip of the longest leaf. The *lil1* seedlings failed to elongate mesocotyls in the dark, and this defect was not restored by BL (**Supplementary Figures 6A, B**). Nevertheless, BL treatment led to a partial rescue, increasing *lil1* shoot length about 1.5-fold compared to untreated *lil1* plants (**Supplementary Figures 6A, C**). Unlike *lil1*, *diz1* seedlings showed a weak etiolation response, but mesocotyls remained much shorter than WT (<1 cm versus ~6 cm WT; **Supplementary Figures 6D, E**). Exogenous BL application had not measurable effect on either mesocotyl or shoot length of the *diz1* (**Supplementary Figures 6D–F**). Together, these observations suggest that the dwarf phenotype of the *diz1* arises from a BR-insensitivity rather than a deficiency in the BR pathway.

### Differential effect of exogenous auxin and gibberellin on the *diz1* mutant morphology

**Figure 3:**
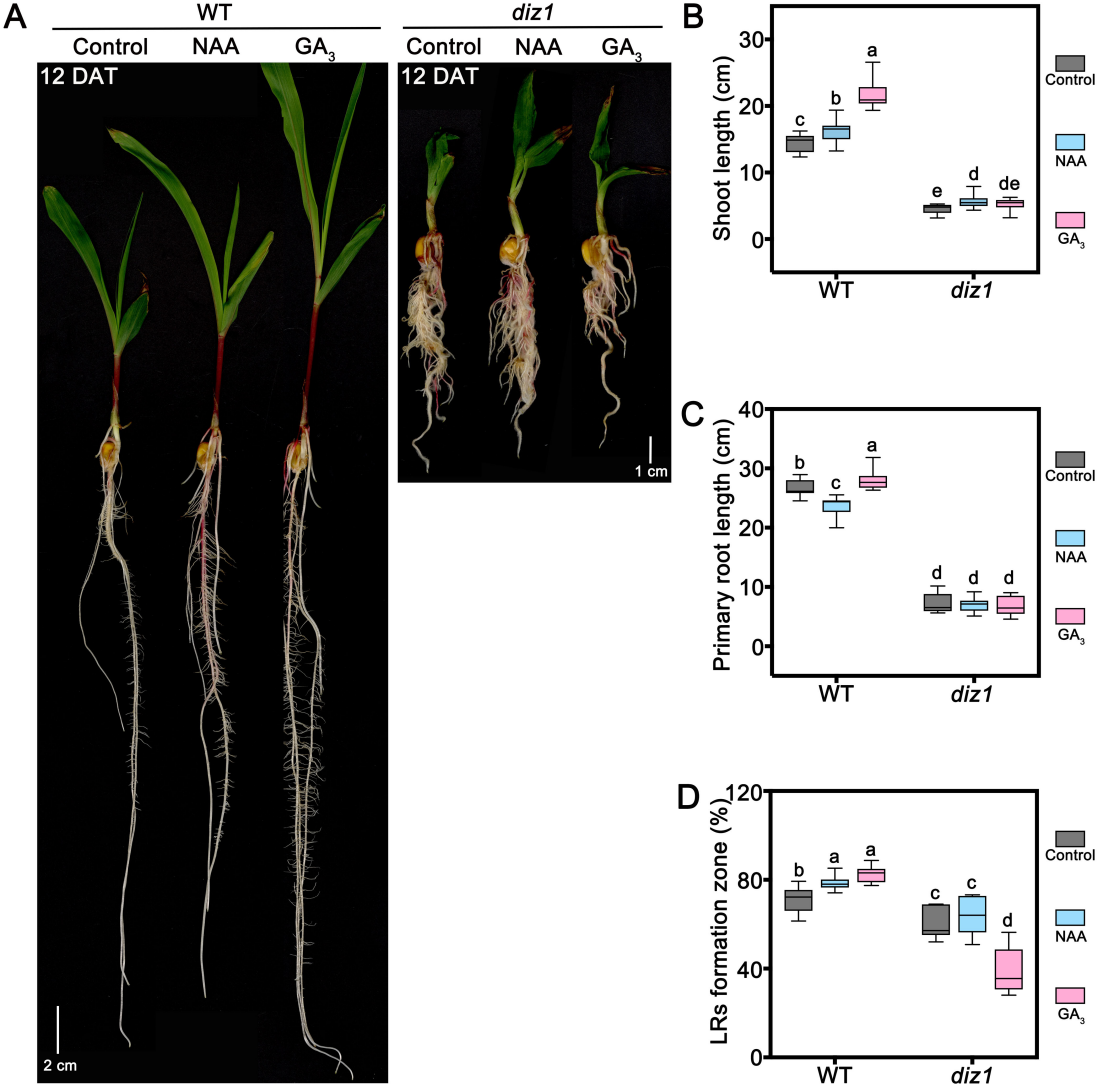
Morphology of the *diz1* mutant with exogenous auxin and gibberellin treatment for 12 days. **(A)** Representative images showing the morphology of WT and *diz1* seedlings at 12 days after treatment (DAT), incubated with or without 1 μM 1-Naphthaleneacetic acid (NAA) and 500 nM gibberellin A_3_ (GA_3_). Scale bars are included in each image. **(B–D)** Shoot length **(B)**, primary root length **(C)** and proportion of the lateral roots (LRs) formation zone **(D)** in WT and *diz1* seedlings at 12 DAT. Data were collected from 15 WT and 7–12 *diz1* seedlings per treatment. The center line of the boxplots indicates the median, the box limits indicate the 25th and 75th percentiles, and the whiskers extend to the minimum and maximum values. Significant differences are presented by letters, based on two-way ANOVA followed by Tukey’s multiple comparison test.

Previous studies have demonstrated that auxin and gibberellin deficiencies can lead to dwarfism in maize (Phillips et al., 2011; Chen et al., 2014). To investigate their effects on *diz1*, we treated mutant seedlings and WT siblings with 1 μM 1-Naphthaleneacetic acid (NAA) or 500 nM gibberellin A_3_ (GA_3_) under hydroponic conditions. At both 9 and 12 DAT, both hormones promoted WT shoot elongation (**Supplementary Figures 7A, B**; **Figures 3A, B**). At 9 DAT, *diz1* shoots were also largely insensitive (**Supplementary Figures 7A, B**). However, extending treatment to 12 days revealed an auxin-specific stimulation of *diz1* shoot elongation, resulting in approximately 25% growth promotion, while GA_3_ remained ineffective (**Figures 3A, B**). In WT, NAA inhibited primary root elongation and GA_3_ promoted it at both 9 and 12 DAT (**Figures 3A,C**; **Supplementary Figures 7A, C**). In *diz1*, neither hormone affected primary root elongation at either time point (**Figures 3A, C**; **Supplementary Figures 7A, C**).

Auxin and GA also regulate lateral root formation. Because *diz1* primary roots develop densely clustered lateral roots (LRs), we measured the proportion of the primary root occupied by the lateral root formation zone (LRFZ). Both hormones increased LRFZ in WT (**Figures 3A, D**; **Supplementary Figures 7A, D**), reflecting their promoting role in lateral root initiation. In *diz1*, NAA had no effect, while GA_3_ reduced LRFZ by 35–37% (**Figures 3A, D**; **Supplementary Figures 7A, D**). Notably, LRFZ% in *diz1* was higher than WT at 9 DAT but lower at 12 DAT (**Figure 3D**; **Supplementary Figure 7D**), indicating early initiation followed by impaired maintenance.

Overall, *diz1* shows differential hormone sensitivities, including a delayed auxin response in shoots, insensitivity to auxin-mediated primary root inhibition, and GA insensitivity in shoot and primary root elongation, with altered lateral root dynamics. These results reveal that *diz1* has impaired hormonal regulation affecting both shoot and root development, with a dynamic shift in sensitivity over time.

### BSR-seq mapped the *diz1* candidate gene to chromosome 2

**Figure 4:**
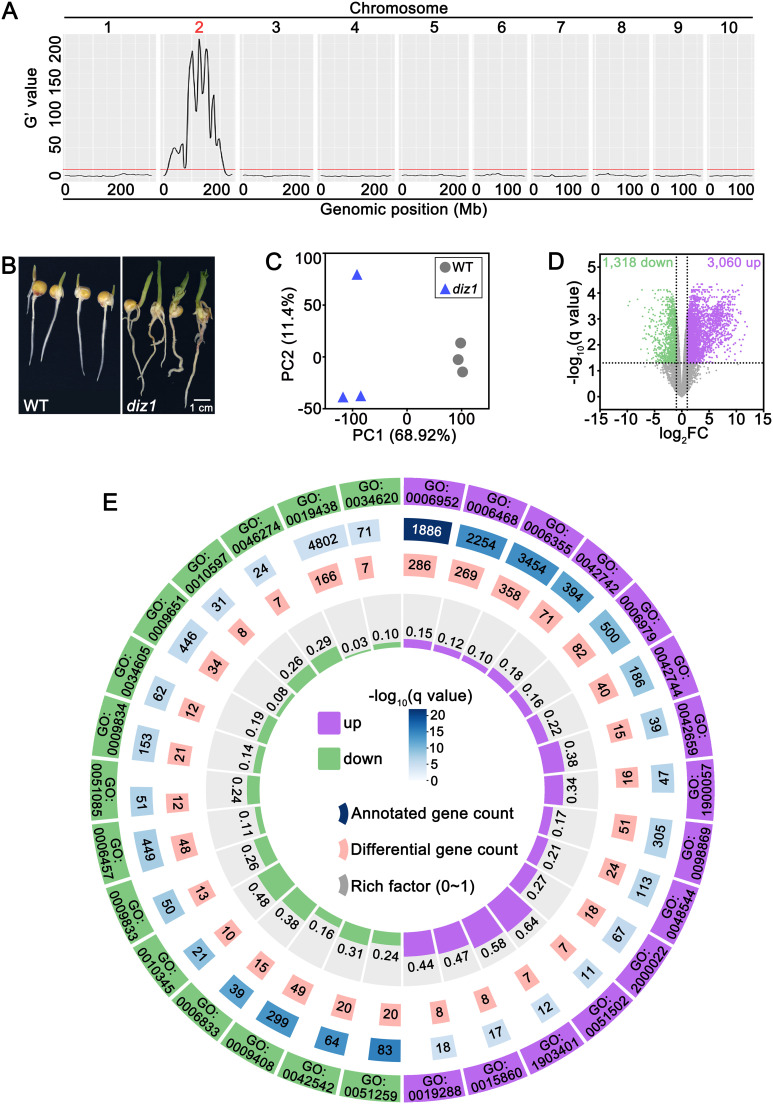
Bulked segregant RNA sequencing (BSR-seq) and RNA-seq analyses in *diz1*. **(A)** BSR-seq mapping confined the candidate gene for the *diz1* mutant on chromosome 2. The red line represents the significant G’ value threshold. **(B)** Exemplary samples of WT and *diz1* seedlings with comparable primary root length (4 cm) used for RNA-seq. Scale bar for both images = 1 cm. **(C)** Principal component analysis of RNA-seq sample libraries from WT (gray circles) and *diz1* (blue triangles). **(D)** Volcano plot depicting differentially expressed genes (DEGs) in *diz1*, identifying 1,318 down-regulated (green) and 3,060 up-regulated (purple) genes. Non-significant genes are shown in gray. **(E)** Functional Gene Ontology (GO) analysis of the 15 most significantly up- and down-regulated biological process (BP) terms in the *diz1* mutant. The outermost circle represents enriched BP terms (purple for up-regulated and green for down-regulated). The second circle displays the total number of annotated genes and the corresponding adjusted *p*-value for each term. The third circle shows the number of differential genes within each term. The innermost circle indicates the enrichment score of each term, and the center contains the legend.

We identified the *diz1* mutant in the *BonnMu* F_2_-family 1-A-0328, which carries 43 germinal *Mu* insertions (Win et al., 2024, Win et al., 2025), making it difficult to directly pinpoint the gene responsible for the observed phenotype. To map the *diz1* locus to a specific chromosome arm, we conducted a BSR-seq analysis using primary roots of a segregating F_2_ mapping population. This population was generated by crossing the inbred line Mo17 with the *BonnMu* F_2_-family 1-A-0328. Based on contrasting phenotypes between WT and *diz1*, we divided the population into two bulked pools (WT and *diz1*), and processed them for RNA sequencing. After sequencing, we aligned the clean reads and called the SNPs. Then, allele frequencies in the WT and *diz1* pool were detected according to Mansfeld and Grumet, 2018, to define the candidate mapping interval of *diz1*. In case of complete linkage of a SNP marker with a mutated gene, only one marker allele is expected to be present in the mutant pool of the mapping population. Plotting allele frequency differences across the genome revealed a significant clustering of SNPs on a genomic interval from 13,876,767 to 215,391,838 bp of chromosome 2, indicating the *diz1* mutation is located on chromosome 2 (**Figure 4A**; **Supplementary Table 2**). Notably, while *brd1*/*lil1* are located on chromosome 1 (Makarevitch et al., 2012; Castorina et al., 2018), further confirming that *diz1* is not allelic to *brd1*/*lil1*.

### Transcriptomic reprogramming in *diz1* highlights developmental dysregulation

To examine transcriptomic changes associated with the mutation in *diz1*, we performed RNA-sequencing on WT and mutant primary roots of identical length (4 cm; **Figure 4B**). As *diz1* displays delayed root development compared to WT, roots of identical length were harvested at different time points (6 DAG for WT and 9 DAG for *diz1*), thereby matching samples by developmental stage rather than by chronological age. Principal component (PC) analysis showed clear genotype-dependent clustering, with WT replicates forming a tight group, indicating high transcriptomic consistency (**Figure 4C**). Mutant samples displayed broader but still genotype-specific dispersion, suggesting greater variability within the *diz1* background. PC1 and PC2 together explained 80.32% of the total variance, with PC1 (68.92%) separating the genotypes and PC2 (11.40%) capturing within-group heterogeneity (**Figure 4C**).

In total, we identified 4,378 differentially expressed genes (DEGs) in *diz1* (**Figure 4D**). Among those, 1,318 DEGs were downregulated and 3,060 were upregulated, revealing a about 2.3-fold bias toward upregulation (**Figure 4D**; **Supplementary Table 3**). To identify enriched biological processes associated with the mutant phenotype, we subjected the DEGs to a functional Gene Ontology (GO) enrichment analysis and visualized the 15 most up- and downregulated biological process (BP) terms (**Figure 4E**; **Supplementary Table 4**). Upregulated terms clustered into plant defense (GO:0006952; GO:0042742; GO:0051502) and oxidative stress responses (GO:0006979; GO:0042744; GO:0098869), along with induction of jasmonic acid-related signaling (GO:2000022; **Figure 4E**; **Supplementary Table 4**). Enrichment of protein phosphorylation (GO:0006468) and regulation of transcription (GO:0006355) reflected broad activation of signaling networks typical for stressed plants (**Figure 4E**; **Supplementary Table 4**), consistent with the growth inhibition and decreased cell viability observed in the mutant. Additional enrichment of recognition of pollen (GO:0048544) and regulation of cell fate specification (GO:0042659) suggested misregulation of reproductive developmental pathways (**Figure 4E**; **Supplementary Table 4**), consistent with the sterile phenotype. The latter GO term could also relate to the reduced primary root growth zone and enhanced root hair elongation (**Figures 1D–G**).

Downregulated DEGs were significantly enriched in BP terms associated with stress tolerance and cellular homeostasis, including response to hydrogen peroxide (H_2_O_2_; GO:0042542), heat (GO:0009408; GO:0034605), salt stress (GO:0009651), and cellular response to unfolded protein (GO:0034620; **Figure 4E**; **Supplementary Table 4**). These patterns suggest a reduced ability of the mutant to cope with H_2_O_2_, heat, salt, and unfolded protein stress, indicating impaired abiotic stress adaptation. Processes related to protein complex oligomerization (GO:0051259), protein folding (GO:0006457), and chaperone cofactor-dependent protein (GO:0051085) were also downregulated (**Figure 4E**; **Supplementary Table 4**), further suggesting destabilized protein and enzyme function. In addition, genes involved in cell wall organization and biogenesis (e.g., GO:0010345, GO:0009833, GO:0009834, GO:0046274) were strongly suppressed (**Figure 4E**; **Supplementary Table 4**), which could weaken structural integrity and explain the increased Evans blue uptake and aberrant cell morphology in the mutant.

Together, these transcriptomic patterns suggest a shift toward constitutive defense activation, accompanied by reduced stress resilience and altered developmental regulation in *diz1*, providing a possible molecular framework for its impaired growth and sterility.

### Increased ROS levels in the *diz1* primary root

**Figure 5:**
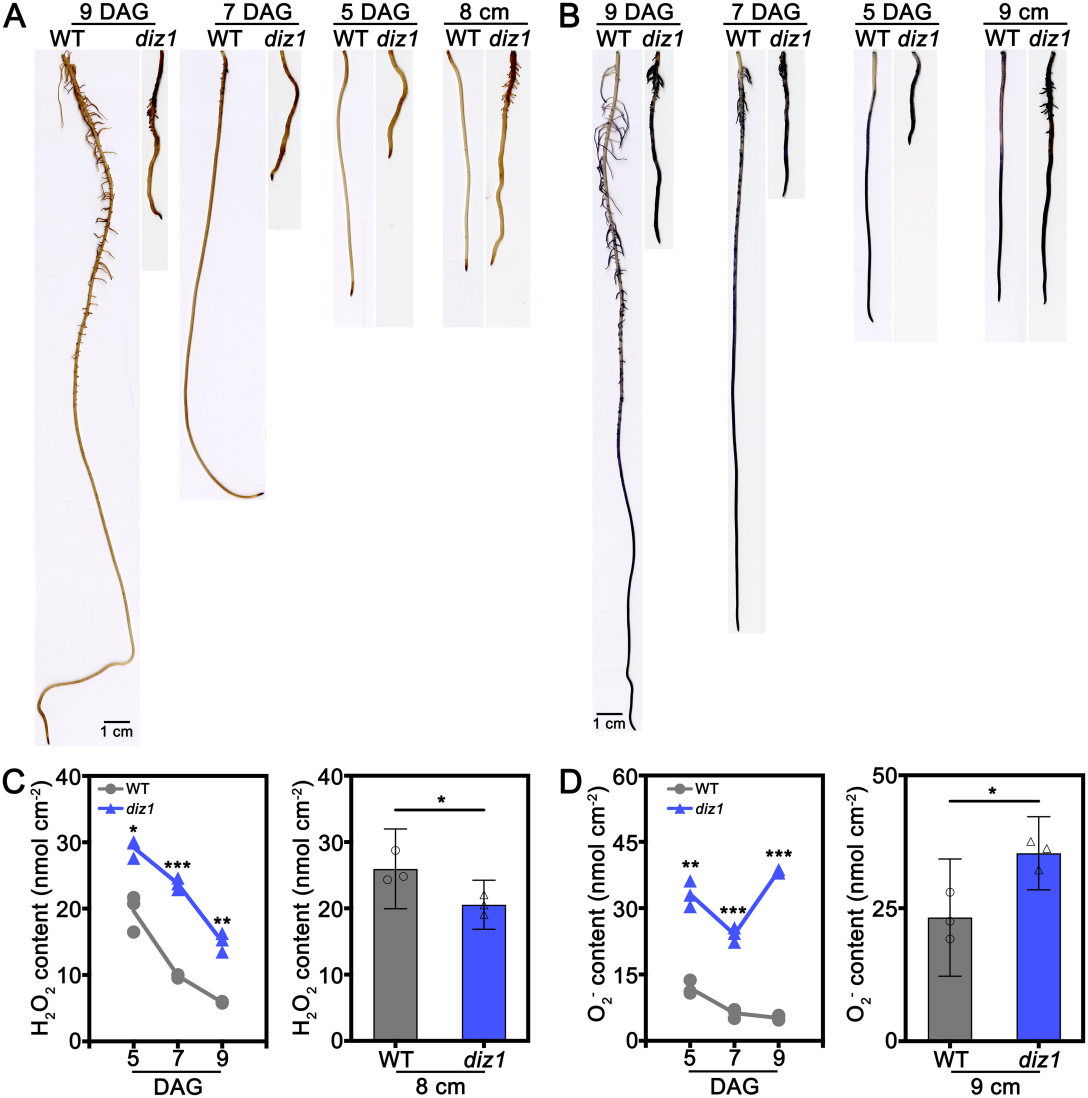
Increased reactive oxygen species (ROS) levels in the primary root of *diz1*. **(A, B)** Primary roots of WT and *diz1* at 5, 7 and 9 DAG, as well as primary roots with a length of 8 cm, stained by 3,3′-diaminobenzidine (DAB) to visualize hydrogen peroxide (H_2_O_2_) accumulation **(A)** and by nitroblue tetrazolium (NBT) to detect superoxide (O_2_^−^) levels **(B)**. Scale bar for all images in **(A, B)** = 1 cm. **(C, D)** Spectrophotometric analysis quantified H_2_O_2_ content in DAB-stained roots **(C)** and O_2_^−^ content in NBT-stained roots **(D)**. Data represent mean ± 95% confidence interval from three independent replicates. Each replicate consists of 3–4 primary root samples. Significant differences are analyzed via a Welch’s *t*-test: *p < 0.05; **p < 0.01; ***p < 0.001.

Given the enrichment of oxidative stress-related genes in the mutant transcriptome, we next examined whether ROS levels were altered. To evaluate hydrogen peroxide (H_2_O_2_) and superoxide (O_2_^-^) accumulation in the primary roots of WT and *diz1*, we used 3,3′-diaminobenzidine (DAB) and nitroblue tetrazolium (NBT) staining, combined with spectrophotometric quantification. DAB forms a dark brown precipitate upon oxidation by H_2_O_2_ (Thordal-Christensen et al., 1997). At 5 DAG, WT primary roots showed DAB staining restricted to the root tip and the apical differentiation zone, whereas *diz1* roots displayed staining that extended throughout the differentiation zone (**Figure 5A**). At later stages, i.e., 7 and 9 DAG, and at equal primary root length (8 cm), DAB staining in WT primary roots remained restricted apically, while in *diz1* it expanded progressively across a larger portion of the differentiation zone (**Figure 5A**). NBT staining, which detects O_2_^-^ through blue-black formazan formation (Doke, 1983), also revealed consistently stronger and more extensive signals in *diz1* from 5 DAG onward. Even at equivalent primary root length (9 cm), *diz1* mutants exhibited darker staining than the WT (**Figure 5B**).

Spectrophotometric quantification supported these staining patterns. When normalized to root surface area, *diz1* accumulated significantly higher levels of H_2_O_2_ and O_2_^-^ than the WT at all comparable developmental stages (**Figures 5C, D**). H_2_O_2_ levels declined over time in both genotypes but remained consistently higher in *diz1*. The apparent exception at equal primary root length (8 cm) reflects differences in developmental timing: WT roots reached 8 cm at 5 DAG, when H_2_O_2_ levels were intrinsically higher, whereas *diz1* required 10–12 DAG, when H_2_O_2_ levels had already declined. Superoxide levels also remained elevated in *diz1*, showing a transient decrease from 5 to 7 DAG followed by an increase at 9 DAG, a pattern that was also observed in primary roots of equal length (**Figure 5D**).

Together, these results show that H_2_O_2_ and O_2_^-^ remain consistently higher in *diz1* than WT under our DAB/NBT assay conditions, consistent with disrupted ROS homeostasis during primary root growth.

### Enhanced lignin deposition in the primary root of *diz1*

**Figure 6:**
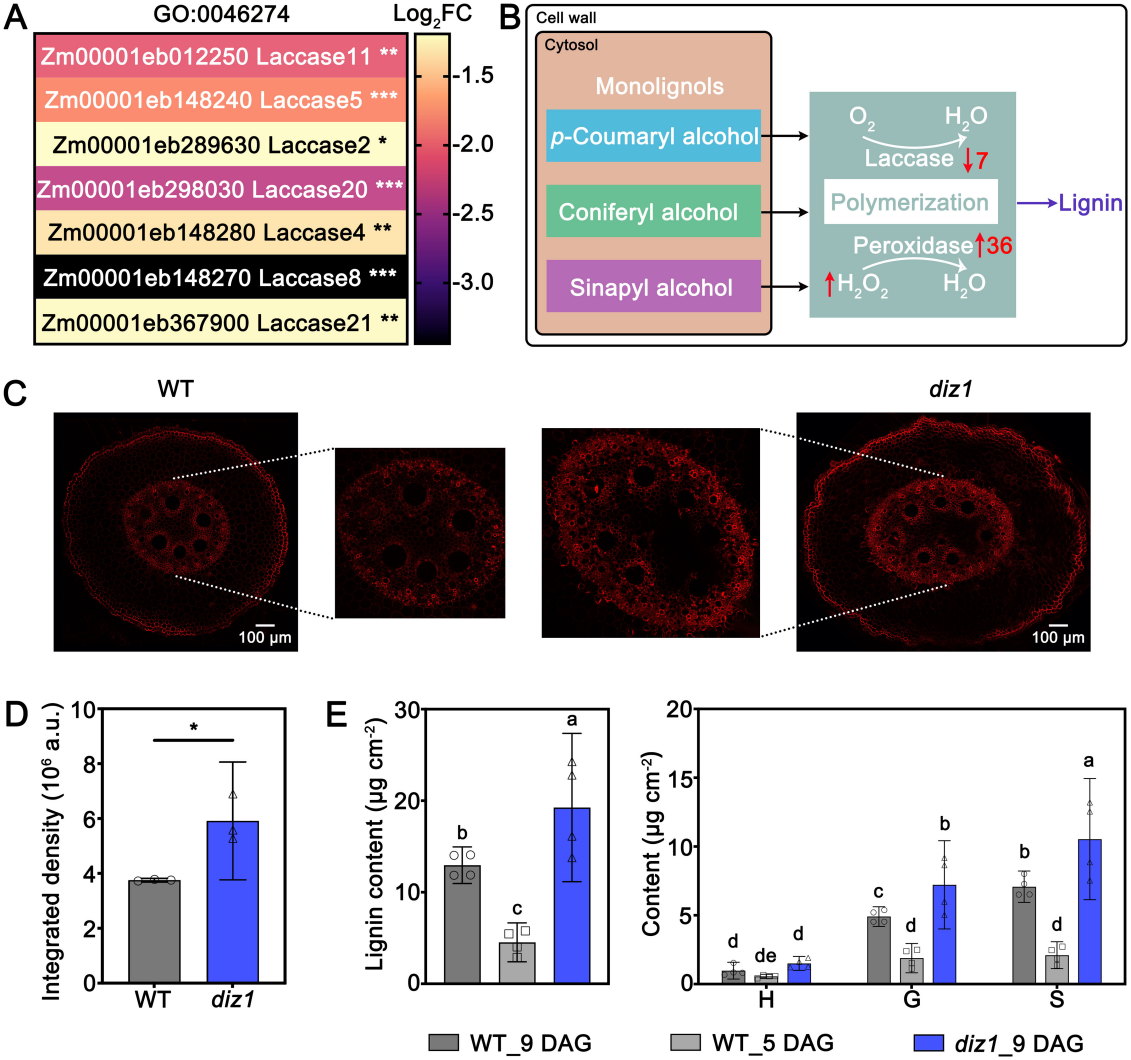
Higher lignin content in the primary root of *diz1* mutant compared with WT**. (A)** Downregulation of seven laccase genes enriched in GO:0046274 (lignin catabolic process) in *diz1*. Asterisks indicate significant differences based on adjusted *p*-value (FDR), *FDR < 0.05; **FDR < 0.01, ***FDR < 0.001. **(B)** Simplified diagram of lignin polymerization from three main monolignols, catalyzed by laccases and class III peroxidases. Red arrows and numbers indicate seven downregulated laccases, thirty-six upregulated peroxidases, and increased H_2_O_2_ levels in *diz1*. **(C)** Basic fuchsin-stained fresh cross-sections of differentiation zone in primary roots at 3 DAG. Images connected by dashed lines indicate zoom-in views of the stele. Scale bars are included in each image. **(D)** Quantification of fluorescence intensity in basic fuchsin-stained sections. Integrated density (mean gray value × area) is shown in arbitrary units (a.u.). Data represent mean ± 95% confidence interval from three biological replicates/genotype. *p < 0.05 (Welch’s *t*-test). **(E)** Lignin quantification in primary roots by thioacidolysis followed by gas chromatography–mass spectrometry (thioacidolysis–GC–MS). Left: total lignin-derived monomers. Right: individual lignin subunits (H: *p*-hydroxyphenyl. G: guaiacyl. S: Syringyl). Data represent mean ± 95% confidence interval from four biological replicates/genotype, each comprising five primary roots. Significant differences (letters) are determined by one-way ANOVA (left) and two-way ANOVA (right) of Tukey’s multiple comparison test.

RNA-seq analysis showed that seven DEGs associated with lignin catabolic processes were significantly downregulated (GO:0046274; **Figure 4E**; **Supplementary Table 4**). Notably, all seven genes encode laccases (**Figure 6A**). Laccases are primarily known for their role in fungal-specific lignin degradation (Wang et al., 2015). Plant laccases share the conserved molecular architecture and reaction mechanism with fungal counterparts, however, in plants, they predominantly function in the oxidative modification and restructuring of lignin polymers (Wang et al., 2015). Both laccases and class III peroxidases catalyze the oxidative polymerization of three main monolignols (*p-*Coumaryl, Coniferyl and Sinapyl alcohols), thereby driving lignin deposition (**Figure 6B**; Peracchi et al., 2024). In contrast to the reduced laccase expression, thirty-six class III peroxidase genes were upregulated and enriched in oxidative stress-related terms (GO:0006979, GO:0042744, GO:0098869; **Figure 4E**; **Supplementary Table 4**). Such activation of peroxidases, together with elevated H_2_O_2_ levels and reduced laccase expression, suggests altered lignin accumulation in the mutant.

To visualize lignin deposition, we stained fresh cross-sections of the differentiation zone of primary roots from WT and *diz1* seedlings with basic fuchsin, a fluorescent dye that specifically binds to lignin (Ursache et al., 2018). Mutant roots displayed brighter and more extensive fluorescence in vascular tissues than WT (**Figure 6C**), and quantitative analysis confirmed significantly higher signal intensity in *diz1* (**Figure 6D**), supporting the hypothesis of increased lignification in the mutant.

We next quantified lignin by thioacidolysis followed by gas chromatography–mass spectrometry (GC–MS) to release lignin-derived *p*-hydroxyphenyl (H), guaiacyl (G), and syringyl (S) monomers from primary roots harvested at 9 DAG. The resulting thioethylated monomers were quantified and normalized to the root surface area. Mutant roots showed a 48 percent higher total yield of monomers than WT (**Figure 6E**). This increase was primarily attributed to elevated levels of G- and S-derived units, which rose by 47% and 48%, respectively (**Figure 6E**). To account for shorter primary roots in the mutant, we further compared lignin levels in primary roots of equal length (5 DAG WT versus 9 DAG mutant). Consistently, the mutant showed a 324% increase in total monomer yield, with H-, G- and S-derived monomers elevated by 170%, 282%, and 400%, respectively (**Figure 6E**). These findings indicate a considerable enhancement in lignification that is independent of developmental timing or root length. Taken together, transcriptional, histological and biochemical analyses support an enhanced lignin deposition in the primary root of *diz1*.

## Discussion

The maize *diz1* mutant is a recessive allele and exhibits a shortened primary root with a reduced growth zone (**Figures 1**, **2**; **Supplementary Figures 1, S2**; **Supplementary Table 1**). We mapped the *diz1* mutation to chromosome 2 (**Figure 4A**) and note that the BSR-seq mapping interval is relatively broad. Moreover, the *diz1* samples exhibit dispersed clustering in the principal component analysis of the RNA-seq data (**Figures 4A, C**), likely reflecting the mixed genetic background of the *BonnMu* F_2_-population (Win et al., 2025). To reduce background complexity and improve mapping resolution, we performed an independent mapping-by-sequencing experiment using a segregating BC4F_2_ population. This confirmed that the causal locus is located on chromosome 2 (data not shown). However, the mapping interval remained large (~52 Mb), likely due to residual genetic heterogeneity despite four backcrosses to B73, preventing the identification of a single candidate gene. Additional backcrossing will therefore be required to further refine the interval. Candidate prioritization by cross-referencing genes within the mapping interval with the 43 *Mu* insertions present in *BonnMu* F_2_-family 1-A-0328 is also limited because the phenotype may result from either an untagged *Mu* insertion or a non-*Mu* mutation (Win et al., 2025).

The 3:1 segregation ratio maintained across selfed generations and backcrosses into both B73 and Mo17 (**Supplementary Table 1**), together with the non-complementation result against *lil1*, are consistent with a single recessive locus. However, each *BonnMu* F_2_-family carries, on average, approximately 53 heritable *Mu* insertions (Win et al., 2024), and monogenic causation therefore requires further validation. Because we have not yet identified an independent *diz1* allele or performed transgenic complementation, we cannot formally exclude the possibility that some phenotypic components described here result from additional co-segregating *Mu* insertions. Accordingly, all conclusions regarding DIZ1 function should be interpreted with this caveat. We initially surveyed previously cloned recessive monogenic dwarf mutants located within the mapping interval on chromosome 2 in maize. Among these, the mutant *d5* (Zm00001eb071070), which encodes *ent*-kaurene synthase involved in GA biosynthesis (Fu et al., 2016), hints for a candidate. However, its expression levels showed no significant difference between *diz1* and WT (**Supplementary Table 3**). In addition, no *d5*-associated SNPs were detected in the BSR-seq analysis of WT and *diz1* bulks (**Supplementary Table 2**). Thus, we find no evidence that *diz1* corresponds to a known dwarfing allele.

Root elongation is a complex process that relies at least in part on cell wall metabolism, cytoskeletal dynamics, energy metabolism, and signaling pathways (Bassani et al., 2004). The defects in root elongation observed in *diz1* resemble those reported for other mutants in which these fundamental processes are disrupted. For example, defects in the actin cytoskeleton cause reduced primary root elongation in the BR deficient *lil1* mutant (Baluška et al., 2001; Castorina et al., 2018). Impaired cell wall formation and reduced cellulose content lead to a loss of anisotropic cell elongation in the root elongation zone of the *carbohydrate partitioning defective28*/*47* (*cpd28*/*47*) mutants (Julius et al., 2021). In rice (*Oryza sativa* L.), impaired protein glycosylation and consequent reduction in auxin levels and cellulose synthesis severely inhibit root elongation in the *mannosyl*-*oligosaccharide glucosidase* (*osmogs*) mutant (Wang et al., 2014), while defective cellulose synthesis results in a short root phenotype of the *endo-1,4-β-D-glucanase* (*osglu3-1*) mutant (Zhang et al., 2012). Consistently, cellulose deficiency also accounts for the shortened roots observed in the barley (*Hordeum vulgare* L.) *hvglu3–1* mutant (Guo et al., 2025). Consistent with these studies, we identified multiple GO terms related to cell wall construction to be significantly downregulated in *diz1*, particularly in cellulose synthesis process (**Figure 4**; **Supplementary Table 4**). Moreover, the reduced root elongation in *diz1* is likely linked to increased lignin deposition (**Figure 6**), a factor known to limit elongation in maize roots (Fan et al., 2006). In this study, the elevated lignin content arises from enrichment of both G- and S-type lignin monomers (**Figure 6E**). G-type lignin enhances wall stiffness through dense cross-linked networks, whereas excessive S-type lignin, despite its linear structure, can further reduce cell wall extensibility (He et al., 2018). Together, these findings suggest that the impaired root elongation observed in *diz1* results from combined alterations in cell wall biosynthesis and lignin deposition. These changes likely compromise cell proliferation in the meristematic zone, anisotropic cell expansion in the elongation zone, and cell differentiation in the differentiation zone. This interpretation is supported by the reduced lengths of the meristematic and elongation zones, as well as the altered cross-sectional cell areas observed across all three developmental zones.

Another feature of *diz1* is its helical twisting growth (**Figure 1**, **2**; (**Supplementary Figures 1–S3**). Organ twisting is often linked to cytoskeletal disorganization. For example, *lil1* shows twisted root cell files due to impaired actin filament organization (Baluška et al., 2001). However, *lil1* primary roots are not directly comparable to those of *diz1*, as their overall root structure remains erect (not shown), unlike the twisted roots observed in *diz1*. Mutants with disrupted cortical microtubule orientation, such as rice *twisted dwarf1-1* (*ostid1-1*, Sunohara et al., 2009), and *Arabidopsis microtubule organization1-1* (*mor1-1*,Sugimoto et al., 2003; Kawamura et al., 2006), *spiral1* (*spr1*, Ishida et al., 2007), *tortifolia1* (*tor1*, Buschmann et al., 2004), exhibit helically twisted growth. In barley, the *hvglu3–1* mutant also shows twisted root shape resulting from defective cellulose (Guo et al., 2025). Consistent with these observations, cytoskeleton- and cellulose-related biological processes were downregulated in *diz1*, including cellulose microfibril organization (GO:0010215) and cellulose biosynthetic process (GO:0030244; **Supplementary Table 4**). Disruption of the cytoskeleton is often accompanied by defects in cell cohesion. Mutants such as *lil1*, *spr1*, and *ostid1–1* not only display twisting but also enlarged intercellular spaces and aberrant cell morphology. In *diz1*, we similarly observed enlarged intercellular spaces and irregular cell shapes (**Figures 2E, F**; **Supplementary Figures 2–S4**). In maize, cell adhesion relies primarily on a hemicellulosic network of arabinoxylan esterified with ferulic acid, while cellulose microfibrils provide the structural framework supporting these linkages (Hatfield et al., 2017). We detected suppression of GO terms related to xylan biosynthetic process (GO:0045492) and cellulose microfibril organization (GO:0010215) in *diz1* (**Supplementary Table 4**), which could weaken cellular linkages. Our hypothesis is that disrupted cytoskeletal organization, coupled with altered cell wall biosynthesis, underlies the twisting growth, irregular cell shapes, and altered cell cohesion observed in *diz1*.

We also observed enhanced root hair elongation in *diz1* (**Figures 1D, E**; **Supplementary Figures 3B, C**). Consistent with this phenotype, RNA-seq revealed an upregulation of maize homologs of *Arabidopsis* root hair regulators, including *root hair defective6* (*rhd6*: Zm00001eb252360), *root hair defective six-like4* (*rsl4*: Zm00001eb374120), *peroxidase73a*/*b* (*prx73a*/*b*: Zm00001eb095550/Zm00001eb058260), and *oxidative signal-inducible1a*/*b* (*oxi1a*/*b*: Zm00001eb079000/Zm00001eb425420), as well as *expansin a7* (*expa7*: Zm00001eb211650), which is also homologous to rice *EXPA17* gene (**Supplementary Table 3**). Transcriptome analyses identified these genes as positive regulators of root hair development (Hey et al., 2017; Zhou et al., 2024). Accordingly, loss-of-function mutants of these genes exhibit reduced root hair length, including *Arabidopsis* mutant *rhd6* (Masucci and Schiefelbein, 1994), *rsl4* (Cui et al., 2024) and rice mutant *osexpa17* (Yu et al., 2011). These regulators act through pathways involving ROS signaling and cell wall remodeling. In line with this, we detect elevated ROS levels and altered cell wall organization in the mutant (**Figures 4**–**6**; **Supplementary Table 4**), further supporting a role for altered signaling in the enhanced root hair elongation observed in *diz1*.

The dwarf phenotype of *diz1* could not be rescued by exogenous application of either GA or BR (**Figure 3**; **Supplementary Figures 6**, **S7**). This lack of morphological response resembles that of known GA- or BR-signaling mutants, including *d8* and *d9* (Winkler and Freeling, 1994; Lawit et al., 2010), *bonzai1* (*bon1*; Jing et al., 2024), and *brassinosteroid insensitive1* (*bri1-RNAi*; Kir et al., 2015), suggesting that loss of DIZ1 function disrupts hormone signal transduction. Consistent with this interpretation, several BR biosynthetic genes, such as *d11* (Zm00001eb080110), *brd1* (Zm00001eb050010) and *constitutive photomorphogenic dwarf* (*cpd*; Zm00001eb196530), which were grouped under the cellular component term GO:0016021: integral component of membrane, were significantly upregulated in *diz1* (**Supplementary Tables 3**, **S4**), a hallmark observed in *bon1* (Jing et al., 2024) and *bri1-RNAi* (Kir et al., 2015). Altered expression of GA-related genes further points to disrupted GA signaling in *diz1*. The GA biosynthetic gene *anther ear2* (*an2*: Zm00001eb021200) was significantly upregulated, while the GA-inactivating gene *gibberellin 2-oxidase1* (*ga2ox1*: Zm00001eb282550) was downregulated (**Supplementary Table 3**), suggesting elevated GA activity in *diz1*. Similar compensatory changes have been reported in *d8*, which accumulates increased levels of bioactive GAs (Fujioka et al., 1988). Together, these transcriptional signatures suggest that loss of DIZ1 affects BR and GA signaling, although whether this reflects a direct regulatory role or a secondary response to developmental perturbation remains to be determined. In addition to altered GA and BR responses, *diz1* shows aberrant auxin responses (**Figure 3**; **Supplementary Figure 7**). In line with this, transcriptome analysis of *diz1* primary roots revealed coordinated changes in genes involved in auxin biosynthesis, transport, and signaling (**Supplementary Table 3**). Several auxin transporters (*pin5c*: Zm00001eb102370, *pin10a*: Zm00001eb158690, *abcb25*: Zm00001eb360840), a biosynthetic gene (*tar2*: Zm00001eb123040), and signaling components (*arf7/29/4/18/16*: Zm00001eb118970/Zm00001eb433020/Zm00001eb067270/Zm00001eb224680/Zm00001eb207450; *iaa2/9/25/32:* Zm00001eb375570/Zm00001eb174180/Zm00001eb432400/Zm00001eb350000) were upregulated, whereas *tar4* (Zm00001eb154790), *yucca3* (Zm00001eb098150), *iaa1* (Zm00001eb271530), *iaa4* (Zm00001eb258220), *iaa31* (Zm00001eb135570) were downregulated (**Supplementary Table 3**). Notably, auxin transporters that are in WT preferentially expressed in shoots and leaves (*pin5c*, *pin10a*, and *abcb25*; Yue et al., 2015), were ectopically expressed in *diz1* primary roots, while the root-enriched *iaa31* (Jiang et al., 2021) was downregulated and the usually low *iaa9* (Jiang et al., 2021) was upregulated in *diz1*. Together, these expression shifts indicate transcriptional reprogramming of auxin pathways and disruption of tissue-specific auxin transport and signaling in *diz1*, explaining the organ-specific auxin responses.

The observed transcriptional reprogramming in *diz1* does not necessarily imply that DIZ1 directly functions in each of the affected pathways. Our finding revealed that treatment with the ROS-inhibitor DPI failed to rescue the shortened root or twisted growth phenotypes of *diz1*, indicating that elevated ROS accumulation is unlikely to be the primary cause of the observed phenotypic defects. The pleiotropic phenotypes of *diz1* may instead represent secondary consequences of a single upstream primary defect, with ROS dysregulation and transcriptional changes reflecting downstream responses. Hormone signaling, redox homeostasis, and cell wall dynamics are interdependent in regulating root growth. Exogenous auxin has been shown to regulate maize primary root elongation by modulating ROS production, which in turn affects cell wall extensibility (Liszkay et al., 2004; Šípošová et al., 2023). Consistent with this model, the dysregulated auxin signaling and persistently elevated ROS levels in *diz1* likely impair the fine-tuned redox control required for cell wall loosening during root elongation. A recent study further supports this interconnection by demonstrating that exogenous BR promotes maize mesocotyl elongation through coordinated regulation of ROS homeostasis and cell wall remodeling, and that combined BR treatment with either IAA or GA produces additive elongation effects (Wang et al., 2025). Together, our findings suggest that *diz1* could coordinate hormonal signaling, ROS balance, and cell wall modification.

Although the causal genetic lesion underlying the *diz1* phenotype remains unknown, our integrated phenotypic, physiological, and transcriptomic analyses provide a strong framework for identifying its genetic basis. The expression patterns highlight candidate pathways that could mediate the pleiotropic effects of *diz1* on growth, development, and constitutive stress responses. Together, these findings deepen our understanding of regulatory networks linking hormonal signaling, redox homeostasis, and cell wall dynamics in plant development, and set the stage for future identification of the causal mutation and its molecular function.

## Data Availability

The datasets generated and analyzed for this study can be found in the Sequence Read Archive (http://www.ncbi.nlm.nih.gov/sra) repositories under the accession numbers PRJNA924739 and PRJNA924225.
