# Supplementary material for: dizzy1 coordinates shoot and root development, constitutive stress responses, and phytohormone signaling in maize
Source: Front Plant Sci. 2026 Aug 5;17:1927522. doi: 10.3389/fpls.2026.1927522 (PMC13486291; doi:10.3389/fpls.2026.1927522)
Supplement: Supplementary file 1 [file DataSheet1.zip › Supplementary Material/Supplementary Figures_S1-S7.pdf]

Supplementary Figures

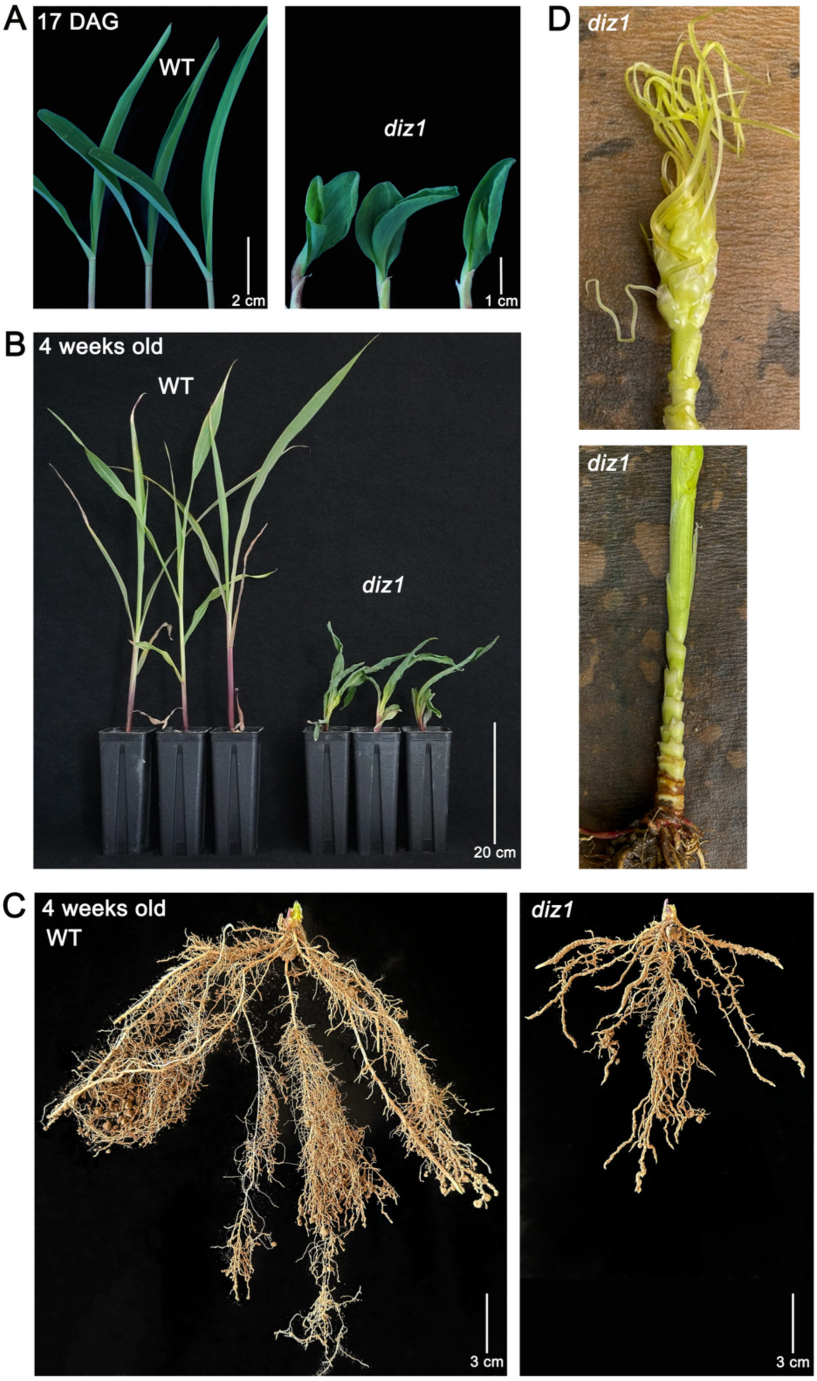

Figure S1. Progressive morphology of the *diz1* mutant throughout development. **(A)** Shoots of WT and *diz1* seedlings at 17 DAG. **(B)** and **(C)** Aboveground morphology **(B)** and root system architecture **(C)** of 4-week-old WT and *diz1* plants grown in pots under controlled conditions in a growth chamber. **(D)** Top: feminized tassel during pollination. Bottom: shortened internodes at the reproductive stage in field-grown *diz1* plants. Scale bars are shown in the respective panels.

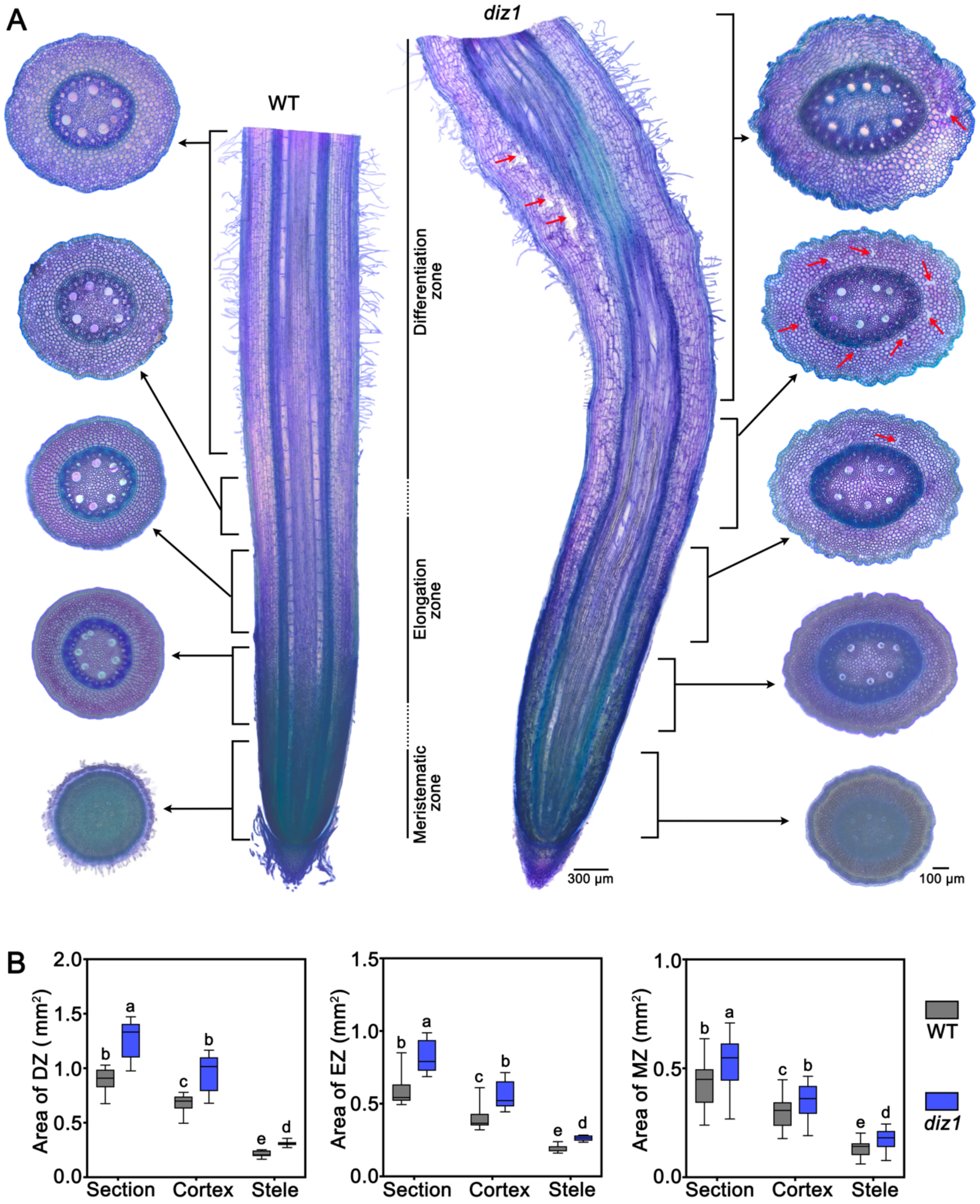

Figure S2. Cellular arrangement in the primary root of WT and *diz1* mutant.

(A) Fresh transverse and longitudinal sections of WT (left) and *diz1* (right) primary roots at 3 DAG, stained with TBO. Black lines/arrows indicate regions of transverse sections; red arrows highlight intercellular spaces in *diz1*. Center lines show root zones, dashed lines: zone transitions. Scale bar for all cross-sections = 100 µm, and for both longitudinal sections = 300 µm. **(B)** Quantification of the entire section area, cortical parenchyma, and stele area from cross-sections of the meristematic zone (MZ),

elongation zone (EZ), and differentiation zone (DZ). Data are from 3–5 sections of 5–7 primary roots per genotype. The center line of the boxplots indicates the median, the box limits indicate the 25th and 75th percentiles, and the whiskers extend to the minimum and maximum values. Significant differences (letters) determined by a two-way ANOVA of Tukey's multiple comparison test.

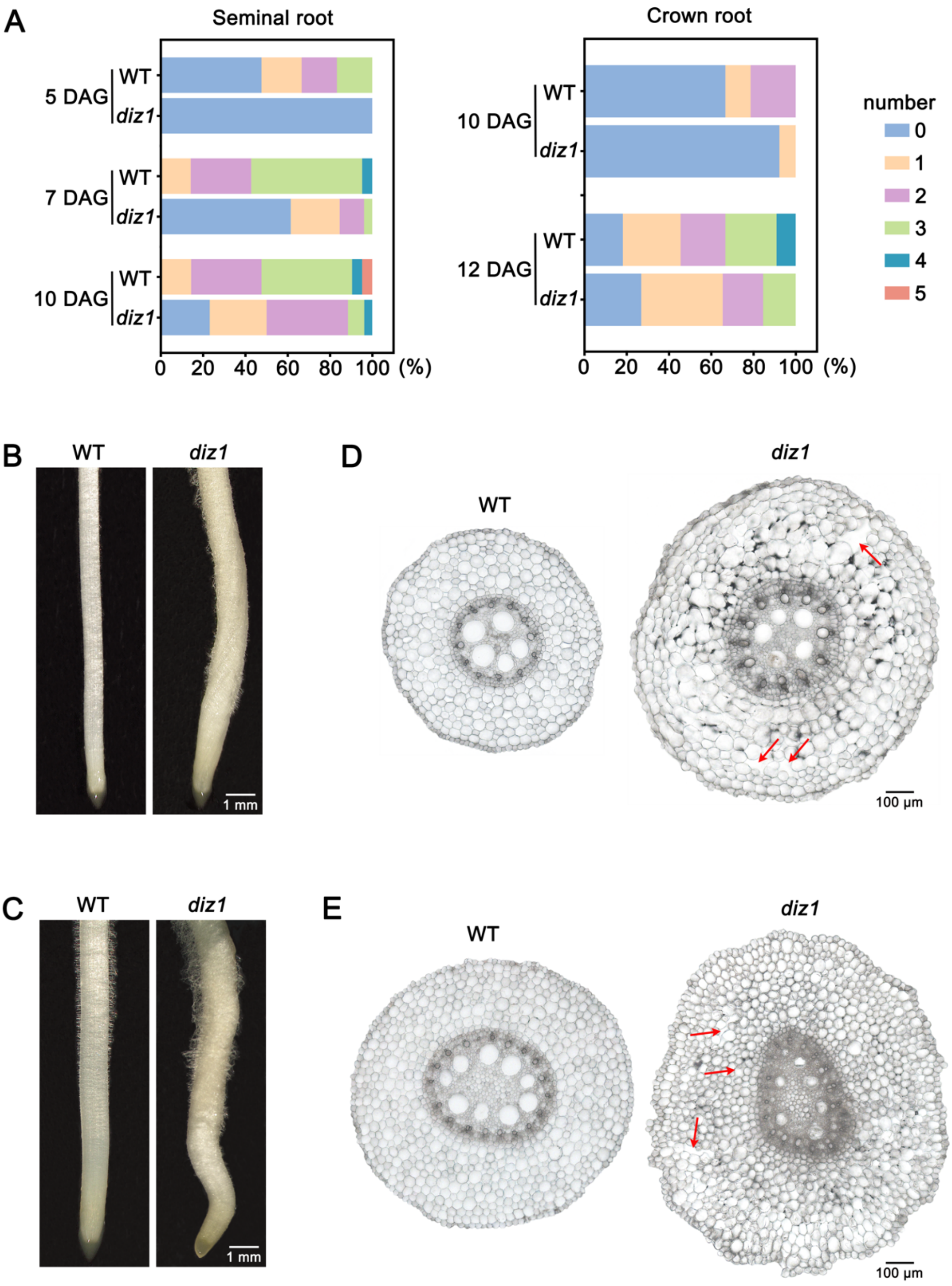

Figure S3. Phenotypic characterization of seminal and crown roots in WT and *diz1* seedlings.

(A) Quantification of seminal root and crown root numbers in WT and *diz1* seedlings at different developmental stages. The number of biological replicates (n) was 42 for WT at 5, 7, and 10 DAG, and 33 at 12 DAG. For *diz1*, n was 26 at 5, 7, 10 and 12 DAG. **(B)** and **(C)** Visualization of seminal roots **(B)** and crown roots **(C)** at 12 DAG using

stereo microscopy. Scale bars for all images in **(B)** and **(C)** = 1 mm. **D** and **E** Fresh cross-sections of the differentiation zone in seminal roots **(D)** and crown roots **(E)** at 12 DAG. Red arrows indicate the intercellular spaces of *diz1* mutant. Scale bar for all images in **(D)** and **(E)** = 100 μm.

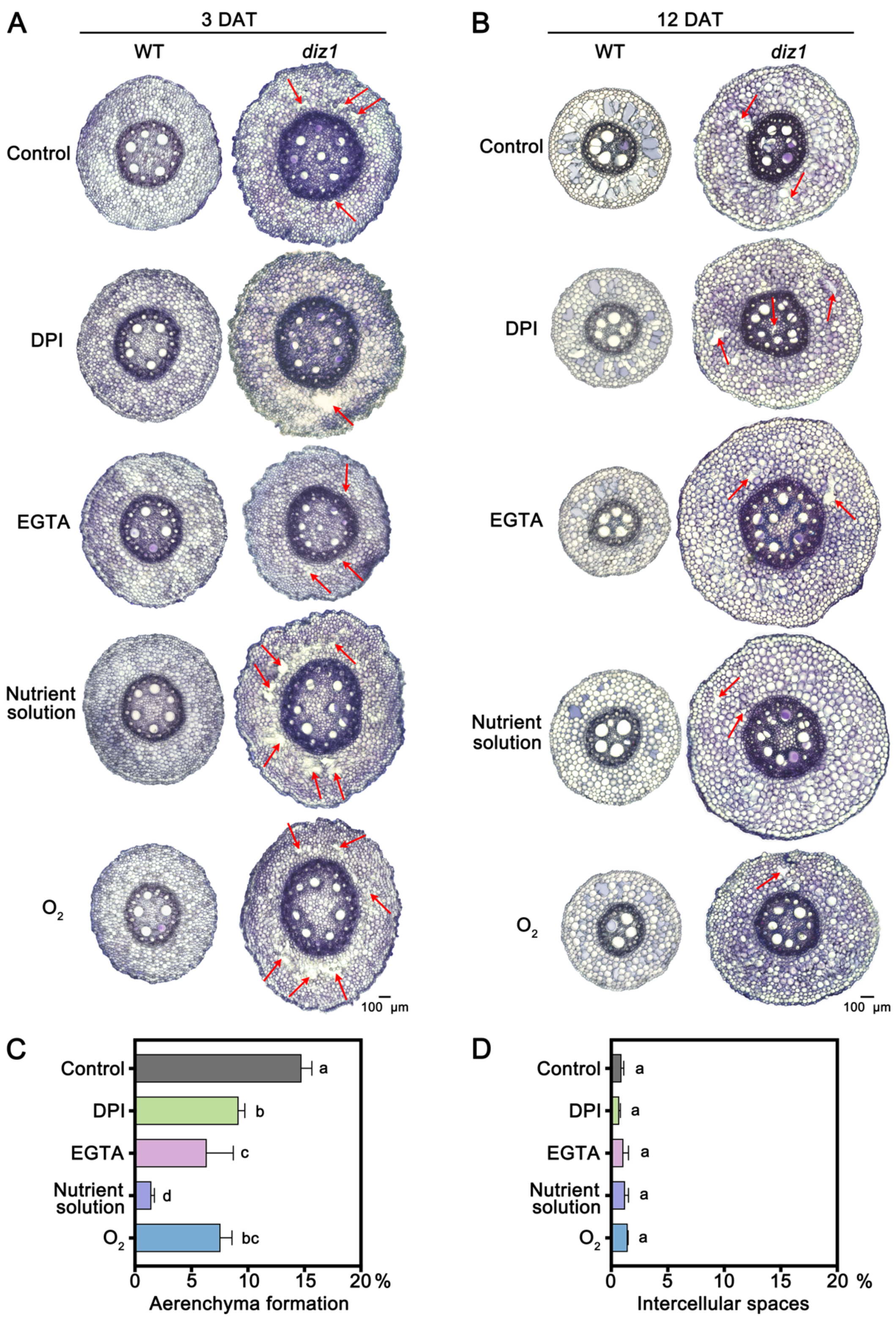

Figure S4. Cortical intercellular spaces in *diz1* are different from lysigenous aerenchyma.

(A) and **(B)** Fresh cross-sections of primary roots with TBO staining under DPI, EGTA, full-strength nutrient solution and O_2 conditions at 3 DAT **(A)** and at 12 DAT **(B)**. Red

arrows indicate visible intercellular spaces in *diz1* mutants. Scale bar for all sections in **(A)** and **(B)** = 100 μm. **(C)** and **(D)** Quantification of aerenchyma formation (presented as the percentage of cross-sectional area occupied by aerenchyma) in WT plants **(C)** and intercellular spaces (presented as the percentage of cross-sectional area occupied by intercellular spaces) in *diz1* mutants **(D)** at 12 DAT. Data are collected from six independent biological replicates per treatment, with each replicate including 3–12 sections. Error bars represent 95% confidence interval. Different letters denote significant differences based on one-way ANOVA with Tukey's multiple comparison test.

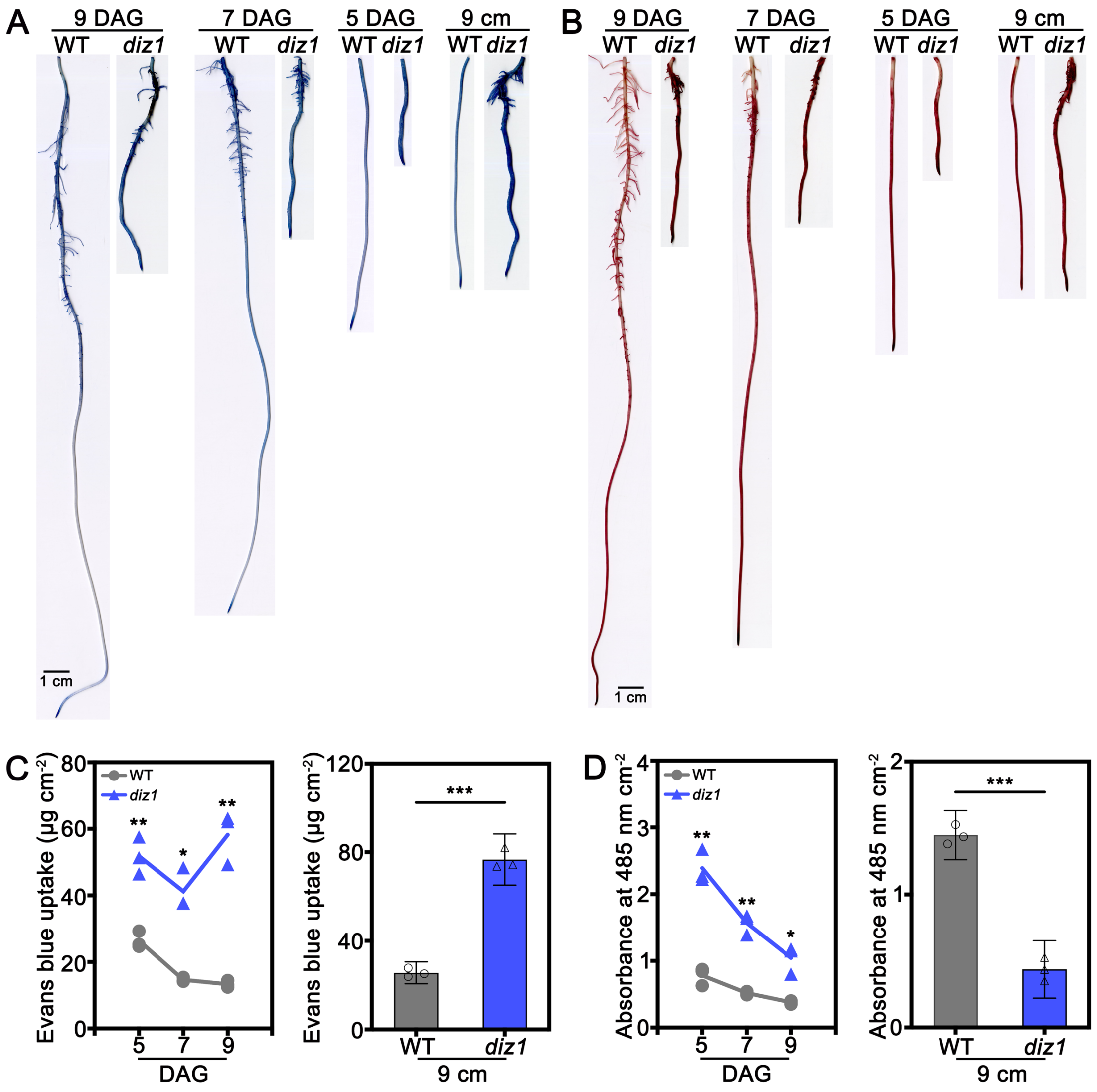

Figure S5. Elevated cell membrane permeability and metabolic activity in the primary root of *diz1*.

(A) and **(B)** Evans blue staining **(A)** and TTC staining **(B)** of comparable primary roots in WT and *diz1* mutants at different developmental stages (i.e., 5, 7, 9 DAG), and same root length (9 cm). Scale bar for all images in **(A)** and **(B)** = 1 cm. **(C)** and **(D)** Spectrophotometric analyses of Evans blue uptake **(C)** and TTC staining absorbance **(D)** in stained primary roots. Data represent mean ± 95% confidence interval from three independent replicates. Each replicate consists of 3–4 primary root samples. Statistical significance is determined using a Welch's *t*-test: *, $p < 0.05$; **, $p < 0.01$; ***, $p < 0.001$.

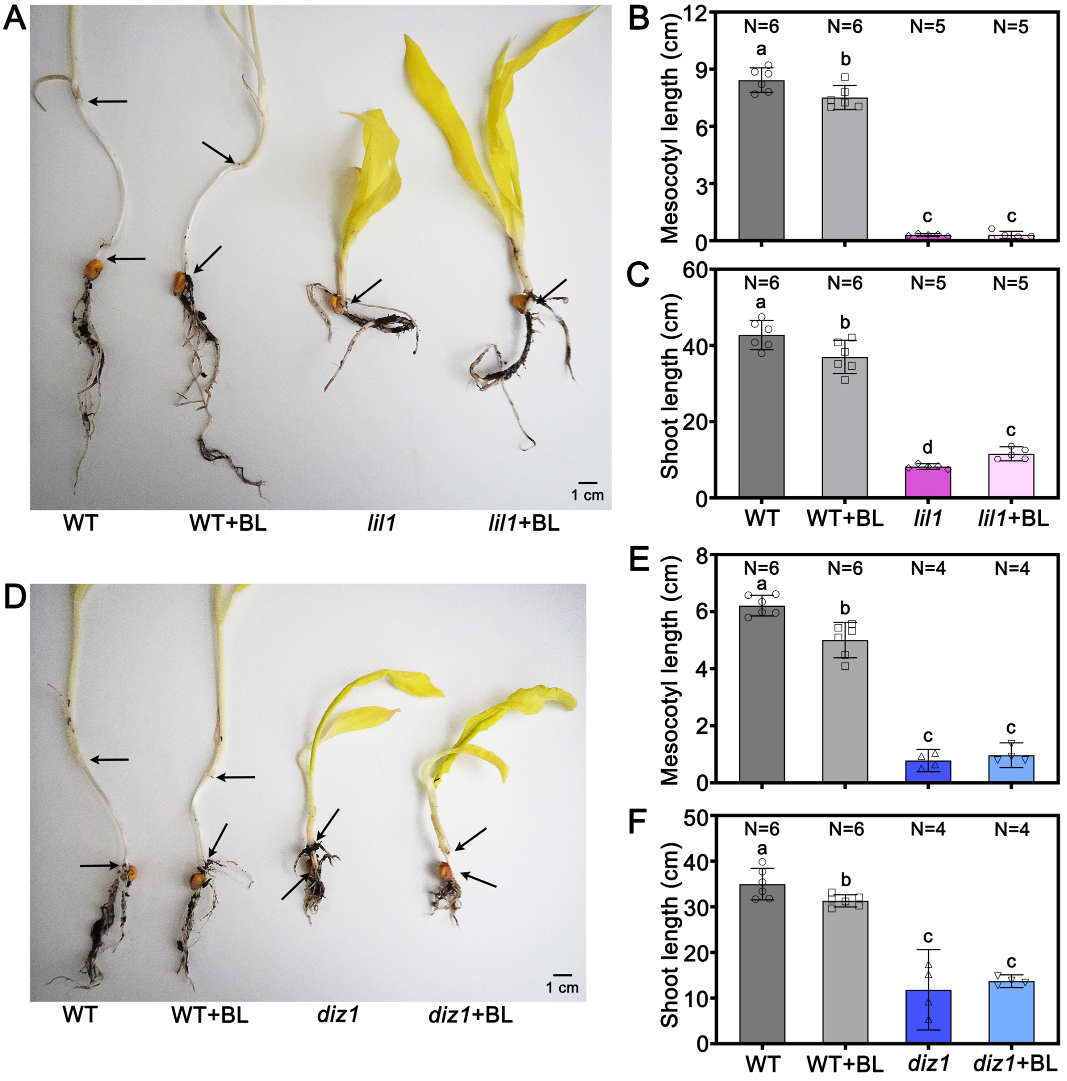

Figure S6. The dwarf phenotype of *diz1* cannot be rescued by brassinolide treatment.

(A) and **(D)** Two-week-old seedlings of *lil1* mutants and their WT siblings **(A)**, and *diz1* mutants and their WT siblings **(D)**, with or without 1 μM BL treatment. Black arrows indicate the position of the mesocotyl. Scale bars = 1 cm. **(B)** and **(E)** The mesocotyl length of *lil1* mutants and their WT siblings **(B)**, and *diz1* mutants and their WT siblings **(E)** after two weeks treatment. **(C)** and **(F)** The shoot length of *lil1* mutants and their WT siblings **(C)**, as well as *diz1* mutants and their WT siblings **(F)** after two weeks treatment. Sample sizes are shown above each column. Error bars represent 95% confidence interval. Significant differences were determined using a two-way ANOVA of Tukey's multiple comparison test, indicated by letters.

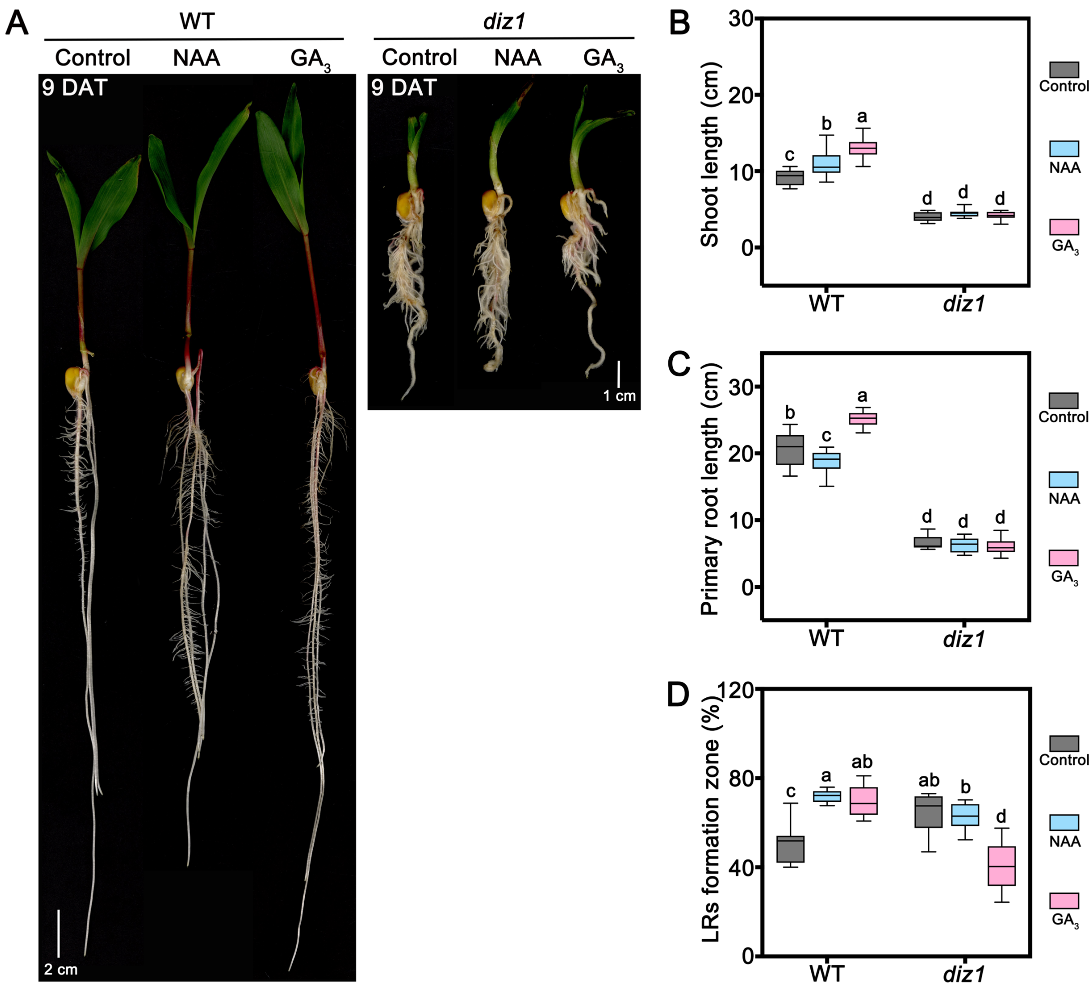

Figure S7. Phenotype of the *diz1* mutant with exogenous auxin and gibberellin treatment for 9 days.

(A) Phenotypes of WT and *diz1* seedlings at 9 DAT with or without 1 µM NAA and 500 nM GA_3. Scale bars are shown in each image. **(B)**, **(C)** and **(D)** Shoot length **(B)**, primary root length **(C)**, and proportion of the lateral roots (LRs) formation zone **(D)** in WT and *diz1* seedlings at 9 DAT. Data represent measurements from 16–18 WT and 7–12 *diz1* seedlings per treatment. The center line of the boxplots indicates the median, the box limits indicate the 25th and 75th percentiles, and the whiskers extend to the minimum and maximum values. Statistically significant differences are indicated by letters, as determined by two-way ANOVA followed by Tukey's multiple comparison test.
